# Mathematical modeling identifies LAG3 and HAVCR2 as biomarkers of T cell exhaustion in melanoma

**DOI:** 10.1016/j.isci.2023.106666

**Published:** 2023-04-13

**Authors:** Richard J. Beck, Sander Sloot, Hirokazu Matsushita, Kazuhiro Kakimi, Joost B. Beltman

**Affiliations:** 1 Division of Drug Discovery and Safety, Leiden Academic Centre for Drug Research, Leiden University, Leiden, the Netherlands; 2 Translational Oncoimmunology, Aichi Cancer Center Research Institute, Nagoya, Japan; 3 Department of Immunotherapeutics, The University of Tokyo Hospital, Tokyo, Japan

**Keywords:** Molecular biology, Bioinformatics, Mathematical biosciences

## Abstract

Cytotoxic T lymphocytes (CTLs) control tumors via lysis of antigen-presenting targets or through secretion of cytokines such as interferon-γ (IFNG), which inhibit tumor cell proliferation. Improved understanding of CTL interactions within solid tumors will aid the development of immunotherapeutic strategies against cancer. In this study, we take a systems biology approach to compare the importance of cytolytic versus IFNG-mediated cytostatic effects in a murine melanoma model (B16F10) and to dissect the contribution of immune checkpoints HAVCR2, LAG3, and PDCD1/CD274 to CTL exhaustion. We integrated multimodal data to inform an ordinary differential equation (ODE) model of CTL activities inside the tumor. Our model predicted that CTL cytotoxicity played only a minor role in tumor control relative to the cytostatic effects of IFNG. Furthermore, our analysis revealed that within B16F10 melanomas HAVCR2 and LAG3 better characterize the development of a dysfunctional CTL phenotype than does the PDCD1/CD274 axis.

## Highlights

• Ordinary differential equation model of T lymphocyte and tumor dynamics *in vivo*
• Model is parameterized with longitudinal multimodal data
• Tumor control primarily achieved with antiproliferative effects and not cytotoxicity
• TIM-3 and LAG3 are biomarkers of the exhausted T cell state

## Graphical abstract

**Figure:**
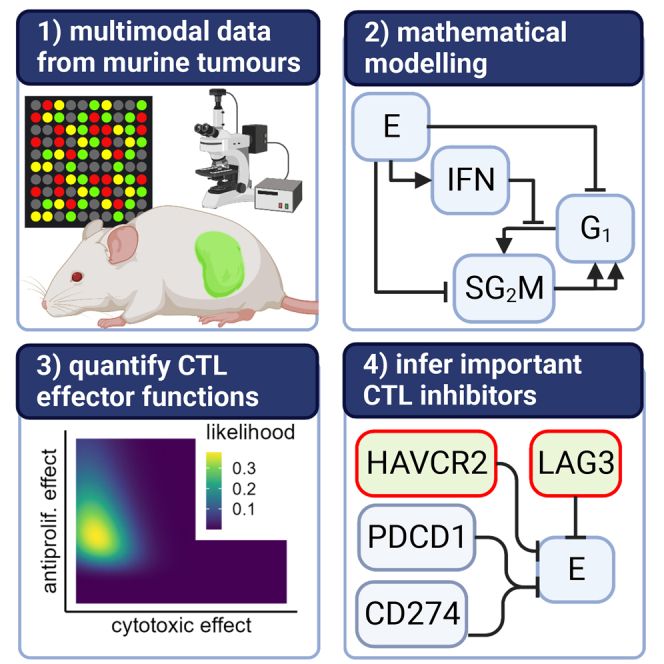

## Introduction

Immunotherapy is an emerging strategy for treatment of cancer, with an ever growing number of immunotherapies having reached clinical trials or been approved already.^1^ Blood cancers were among the first to be successfully treated with immunotherapy^2^; to date solid tumors have proved to be more challenging. Despite this, several treatments are already available for solid tumors and many more are under trial.^3^ Despite some success with immunotherapy so far, there remains a pressing need for greater mechanistic understanding of immune cell interactions within solid tumors. Such understanding may help expand the scope of immunotherapies to different cancers, identify biomarkers to predict patients benefiting from immunotherapy,^4^ optimize dosing schedules for immunotherapies,^5^^,^^6^ or identify combination therapeutic strategies.^7^ Computational models are a useful tool to develop such understanding since they can link data from different sources and make quantitative predictions for what we should expect under different conditions.

CD8^+^ cytotoxic T lymphocytes (CTLs) are key players in the anti-cancer immune response, and many immunotherapy strategies are focused on them. Prominent examples are blockade of inhibitory receptors such as programmed cell death protein 1 PDCD1 (common alias: PD-1, encoded by the *Pdcd1* gene) expressed on CTLs to “remove the brakes” on the immune response^8^^,^^9^ or adoptive transfer of engineered T cells.^10^^,^^11^ Understanding the functioning of CTLs inside tumors is of foundational importance for rational immunotherapy design. Secretion of the cytokine interferon-γ (IFNG) is a hallmark of activated CTLs, yet due to its pleiotropic effects the exact effects of this cytokine in solid tumors remain poorly understood. Some have even noted the “paradoxical” role of IFNG in tumor progression,^12^ paradoxical in the sense that IFNG can have both pro-tumor and anti-tumor effects. Among the pro-tumor effects, IFNG can lead to recruitment of suppressive cells like regulatory T cells or myeloid-derived suppressor cells (MDSCs) or induce expression of immune checkpoint ligands like programmed death-ligand 1CD274 (common alias: PD-L1, encoded by the *Cd274* gene) on tumor cells.^13^^,^^14^ Among the anti-tumor effects, IFNG can aid in the recruitment of innate immune effectors, kill tumor cells, or exert antiproliferative effects on tumor cells.^15^^,^^16^^,^^17^

Despite the recognition that IFNG has both pro-tumor and anti-tumor effects on cancer cells, these effects have not been quantified in great detail. With respect to anti-tumoral effects, we have previously used computational models to demonstrate how an antiproliferative effect mediated by cytokines could potently limit tumor progression since through cytokine signaling CTLs can control many tumor cells, stalling tumor growth and buying time for killing of tumor cells by CTLs.^18^^,^^19^ However, in our previous modeling work, no direct data linking the proliferation of tumor cells to cytokine levels inside the tumors were available. Indeed, antiproliferative effects of IFNG are mediated by inhibitors of cyclin-dependent kinases, which result in arrest of tumor cells at the G_1_ phase of the cell cycle, as shown in a variety of cell lines.^15^^,^^16^^,^^17^ With respect to quantification of pro-tumoral effects of IFNG, it is currently unclear what is the relative importance of various T cell exhaustion markers that limit the immune response against cancer. Although the clinical success of immune checkpoint blocking antibodies demonstrates the significance of T cell exhaustion in tumor progression, there remains an urgent need for mechanistic and quantitative insight into exhaustion pathways in order to identify effective treatment combinations and biomarkers predicting response.

In the current study we sought to gain a quantitative understanding of the role of both the dynamics of IFNG-mediated antiproliferative effects and the exhaustion marker dynamics. Therefore, we utilize data from Matshushita and coworkers where they explicitly explored the antiproliferative effects of IFNG following *in vivo* T cell adoptive transfer by means of a cell cycle sensor in B16F10 melanoma.^15^ Moreover, we exploited dynamic mRNA expression data from that study, allowing us to longitudinally quantify the expression of various exhaustion markers throughout adoptive transfer treatments. Although mathematical models of T cell exhaustion have been developed,^20^^,^^21^ these have so far focused only on PDCD1- or cytotoxic T-lymphocyte-associated protein 4 **(**CTLA-4)-mediated inhibition and not on other checkpoints available in our data, thus necessitating development of a new mathematical model here. As a first step to quantify the importance of IFNG in tumor control relative to the canonical killing functions of CTLs, we developed an ODE model, which integrated data from Matshushita et al.^15^ to arrive at a coherent, quantitative description of the intratumoral activities of CTLs and their interactions with the tumor following adoptive transfer. Consistent with our previous work on this topic,^19^ our model predicted that the cytotoxic effects of CTLs were small and that cytostatic effects of IFNG were responsible for almost all of the observed difference in tumor growth between CTL-treated versus untreated tumors. As a second step, we extended our analysis and modeling to integrate the dynamics of various exhaustion markers as potential explanation for the short window of IFNG production, with CTLs losing the ability to produce IFNG within days of CTLs infiltrating the tumor. Markers of CTL exhaustion such as HAVCR2 (encoded by the *Havcr2* gene) and LAG3 (encoded by the *Lag3* gene) increased over this period, suggesting that CTLs had become exhausted inside the tumor. In contrast to HAVCR2 and LAG3, the dynamics of PDCD1 and CD274 did not coincide with the dynamics of CTL exhaustion, suggesting a relatively minor role for PD-1/PD-L1 as determinants of CTL exhaustion in the B16F10 melanoma model, at least at late stages of anti-tumour immune responses.

## Results

### Presence of CTLs correlates with cell-cycle arrest in tumor cells

**Figure 1:**
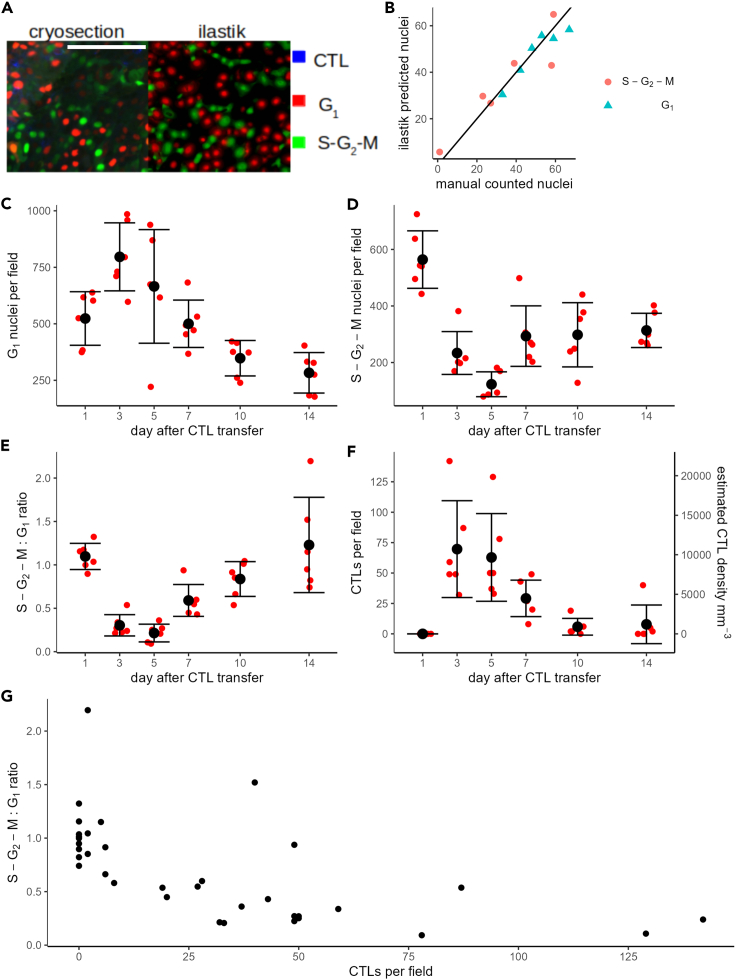
Dynamics of tumor cell-cycle arrest correlates closely with CTL presence (A) Comparison of cryosection image (left), with probabilities predicted by ilastik (right). G_1_ and S-G_2_-M phase nuclei are shown (respectively) in red or green. CTLs appear blue in the cryosection image and were not quantified using ilastik. Image shown is an example of a subregion (scale bar indicates 100 μm) of one complete cryosection (750 × 550μm), which was used for training the classifier. (B) Number of nuclei in cryosection subregions used for training the classifier, comparing manually counted (horizontal axis) nuclei with the ilastik estimate (vertical axis). (C and D) Results of automated quantification of the number of B16F10 nuclei in the G_1_ phase (C) or in the S-G_2_-M phases (D), applied to the full set of cryosection images. E) Ratio of cells in S-G_2_-M:G_1_ phases determined from the automated density estimation results (shown in C and D). (F) Number of CTLs manually counted in each cryosection. Each small red point in C-F represents the total number of cells counted per cryosection, large black points and error bars are (respectively) mean +/− standard deviation. (G) Comparison of the number of CTLs (horizontal axis) with the tumor cell S-G_2_-M:G_1_ ratio (vertical axis) for each available fluorescent image.

A previous study employed a Fluorescence Ubiquitin Cell Cycle Indicator (FUCCI) cell-cycle sensor to show that adoptive transfer of CTLs induced G1-phase cell-cycle arrest of B16F10 tumor cells in an IFNG-dependent manner;^15^ however, the temporal evolution of this arrested state and correlation with the number of tumor-infiltrating CTLs was not explicitly quantified. Therefore, we exploited previously unquantified images from the same study, taken at multiple time points after CTL transfer, to estimate the number of tumor-infiltrating CTLs and B16F10 tumor nuclei. To quantify the number of B16F10 nuclei in either the G_1_ phase or in the S-G_2_-M phases at different time points after CTL transfer, we developed automated pipelines using the ilastik^22^ cell density estimation tool (see STAR Methods). Comparison of the ilastik predictions for small subregions of sample images (Figure 1A) to manual counts made for the same images demonstrated that our pipeline was reliable (Figure 1B). Moreover, our estimated densities of G_1_ phase (Figure 1C) or S-G_2_-M phase (Figure 1D) on day 3 were comparable to those in the study of Matsushita et al.,^15^ as were the ratios of cells in G_1_:S-G_2_-M phases (Figure 1E). Due to their irregular morphology, detection of CTLs was difficult to automate, so we instead performed a manual count of the number of CTLs across all images (Figure 1F). We found a strong negative correlation between the number of CTLs and the G_1_:S-G_2_-M ratio in the sample images (Figure 1G), with a Pearson’s correlation coefficient of −0.60 (95% confidence interval between −0.78 and −0.33) allowing us to reject the null hypothesis of no correlation (p = 0.00015). In summary, the G_1_ cell-cycle arrest following CTL transfer lasted for up to 5 days, and its temporal dynamics were closely linked to the presence of CTLs inside the tumor.

### Tumor cell-cycle arrest correlates with tumor growth reduction

**Figure 2:**
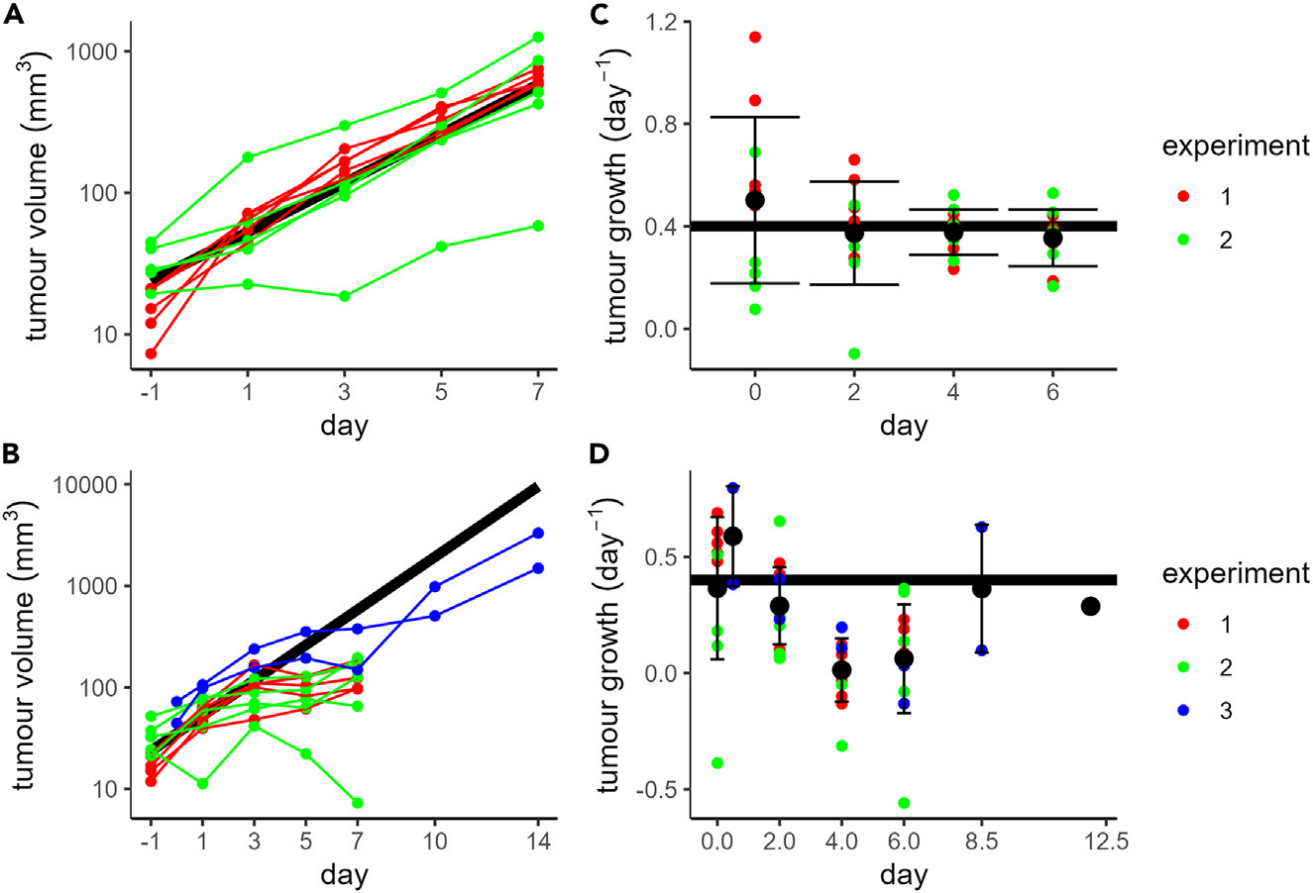
Tumor volume dynamics corresponds to G_1_ cell-cycle arrest and CTL presence (A) Tumor volume progression from 2 different experiments (n = 5 mice per experiment) without CTL treatment. (B) Tumor volume progression from 3 different experiments (n = 5 mice in experiments 1&2, n = 2 mice in experiment 3) with CTLs transferred on day 0. (C) Untreated tumor growth rate estimates for each mouse taken across each of the measurement intervals shown in A. (D) Tumor growth rate estimates for each mouse receiving CTL transfer on day 0. Solid black lines in A-D show results of fitting an exponential growth model (g = 0.4 day^−1^) to the untreated data (A,C) and are shown alongside CTL-treated data (B,D) for comparison. Black points and error bars in C-D represent (respectively) mean +/− standard deviation of all points. Points in C-D are shown at the midpoint of the interval over which they were estimated. Colored dots in all panels indicate the independent experiments consisting of multiple mice.

To check if the temporary G_1_ cell-cycle arrest was consistent with tumor volume progression, we also incorporated tumor volume measurements into our analysis. An exponential model of tumor growth was sufficient to describe tumor progression over the studied interval (Figure 2A), i.e., within the observed range of tumor sizes, there was not yet any indication for a potential carrying capacity limiting tumor growth. Note that although there was considerable variability between tumor growth rates of individual mice, the population estimate (0.4 day^−1^) was similar to the mean of the growth rates estimated from individual mice (Figure S1). Volume estimates were available from three separate experiments with CTL treatment (Figure 2B). We noted some minor yet apparent systematic differences between experiments. For instance, almost all volumes recorded on day −1 were larger in one of the biological replicates (compare red and green points in Figures 2A and 2B, day −1). Despite these minor discrepancies, the broad pattern of tumor progression was similar across replicates, with substantially arrested growth between days 3–7. Nevertheless, such systematic differences between experiments could potentially distort our results, for example, because the switching from 12 mice to 2 mice between measurements going from days 7–10 (Figure 2B) would artificially introduce a period of tumor growth above even the untreated growth rate into our data. To avoid this issue, we converted the data into estimates of the tumor growth rate between measurement intervals for both the data without CTL transfer (Figure 2C) and those with CTL transfer (Figure 2D). For the experiments where CTLs were transferred, this resulted in consistent values between experiments and allowed us to safely incorporate the additional measurements from the 2 mice that were recorded up until day 14. From this analysis, reduced tumor growth was apparent between days 3–7 (Figure 2D; points centered on days 4 and 6) but growth recovery in the measurement interval between days 7–10 (Figure 2D; point centered on day 8.5). Therefore the period of tumor growth reduction was coincident with the period of G_1_-phase tumor cell-cycle arrest (Figure 1E) and by extension also coincident with the presence of CTLs within the tumor (Figure 1F)

### Loss of IFNG production precedes loss of CTLs from tumors

**Figure 3:**
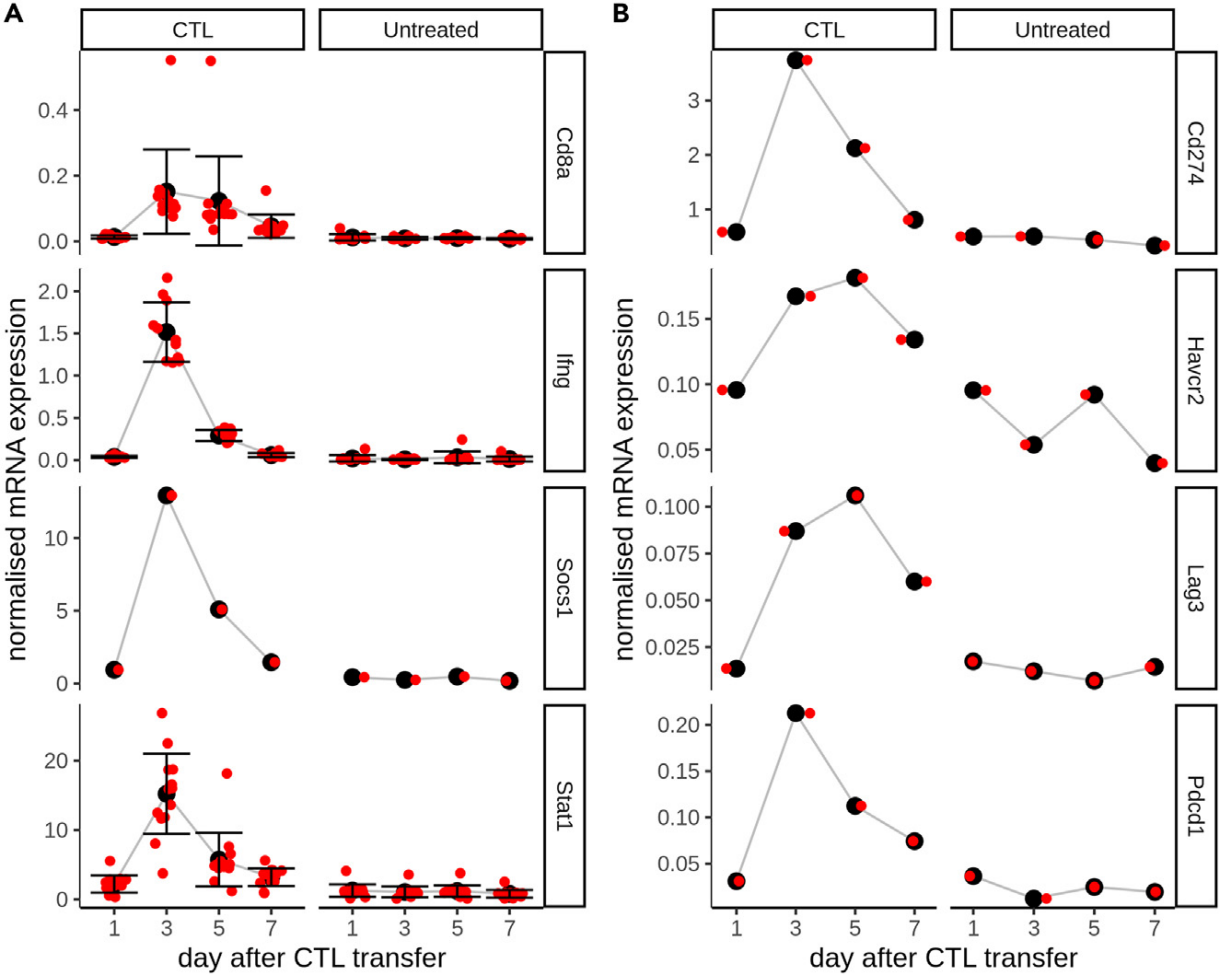
Dynamics of selected genes from microarray dataset (A) Comparison of dynamics of probes specific for Cd8a (row1), Ifng (row 2), Socs1 (row 3), or Stat1 (row 4) between CTL-treated and untreated mice (across columns). Black points and error bars in A represent (respectively) mean ± s.d for all probes at a given time point. (B) Comparison of dynamics of probes specific for mRNA coding PDCD1 (Pdcd1) and CD274 (Cd274), HAVCR2 (Havcr2), and LAG3 (Lag3) (along rows), between CTL-treated and untreated mice (across columns). Red points in A-B represent expression values after normalization at the 75th percentile.

IFNG secreted by CTLs was the putative agent which led to cell-cycle arrest and the transient reduction of tumor progression in our studied data.^15^ As a proxy for IFNG levels inside the tumor, we used mRNA expression data recorded within the same experiments as the previously analyzed image (Figure 1) and volume progression data (Figure 2). We found that *Cd8a* transcription dynamics (Figure 3A, row 1) matched the CTL dynamics measured in the images (Figure 1F), indicating agreement between the transcriptomics data and the imaging data with respect to CTL abundance. However, the dynamics of *Ifng* transcription appeared much different from those of the CTLs (Figure 3A, row 2). *Ifng* transcription peaked sharply on day 3 after CTL transfer but had dropped sharply by day 5 and returned to basal levels on day 7, when CTLs still remained inside the tumor. To verify the dynamics of *Ifng*, we also checked *Stat1* and *Socs1* (Figure 3A, rows 3–4), which are downstream of the IFNG receptor^23^ in the IFNG signaling pathway. These followed very similar dynamics to *Ifng* mRNA, lending support to the idea that the *Ifng* mRNA expression data were a suitable proxy for IFNG signaling dynamics inside the tumor.

We hypothesized that the difference in dynamics between CTLs and *Ifng* transcription was due to a gradual CTL exhaustion inside the tumor, leading to a loss of their effector functions. Exhausted T cells display hierarchical loss of effector functions including proliferative ability, capacity to kill target cells, and secretion of cytokines such as IFNG.^24^^,^^25^ Several genes are associated with the exhausted T cell state,^26^^,^^27^ and as T cells become progressively more exhausted, they express a greater diversity of inhibitory receptors.^25^ Indeed, we could identify transcripts for a number of well-described immune checkpoint molecules in the mRNA dataset, including PDCD1, its ligand CD274, LAG3, and HAVCR2 (Figure 3B). Overall, our analysis suggests that the pulse of IFNG production remains brief despite CTLs still being present within the tumor and might be due to development of an exhausted phenotype among the transferred CTLs.

### IFNG transcription dynamics are compatible with G_1_-phase tumor cell-cycle arrest

**Figure 4:**
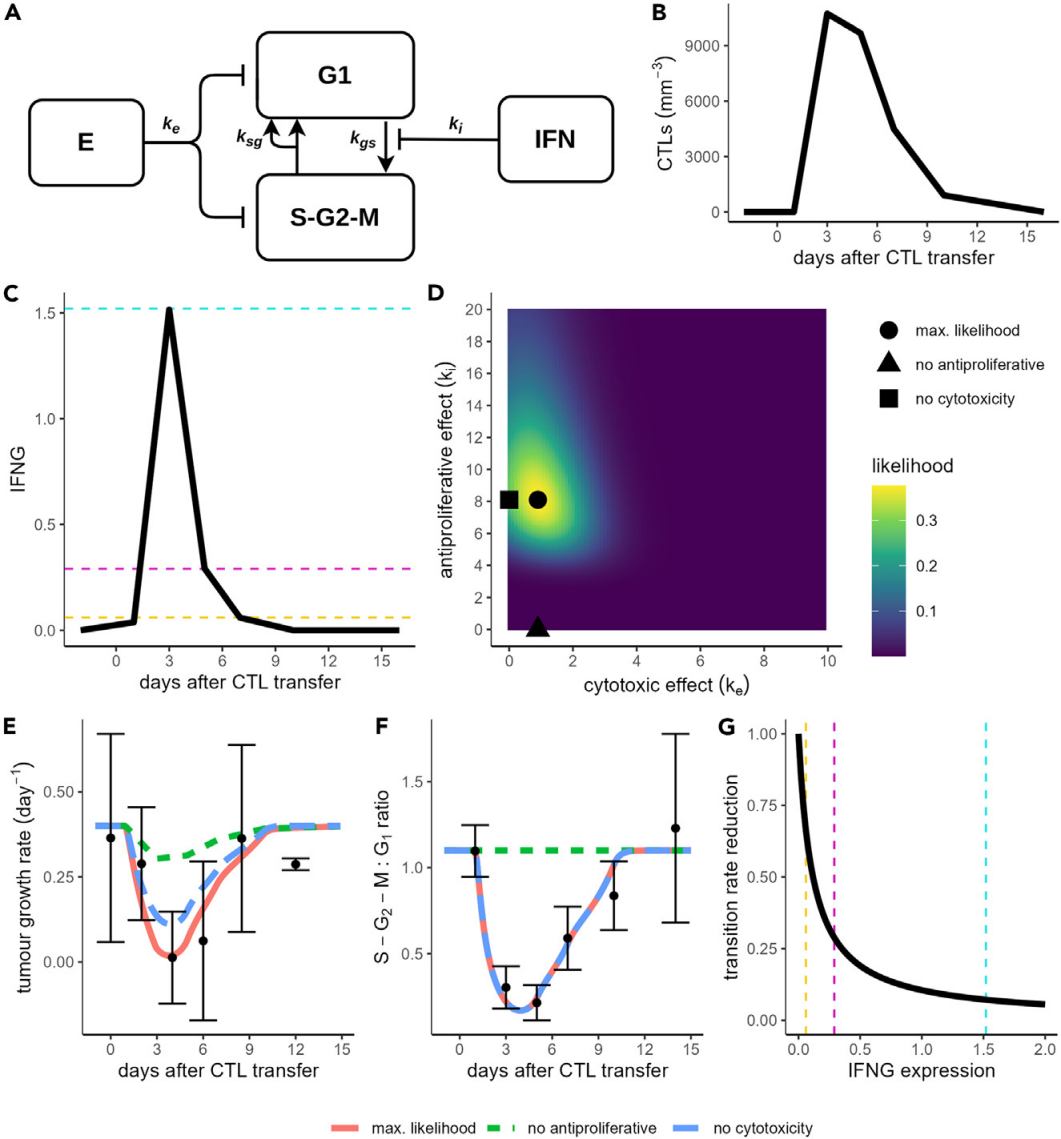
Compatibility of *Ifng* transcription dynamics with tumor cell-cycle arrest (A) Schematic for ODE model to compare CTL-mediated killing and IFNG-mediated antiproliferative effect in B16F10 melanoma. (B and C) Linear interpolation of mean CTL density (B) and mean *Ifng* expression (C). (D) Heatmap showing likelihood for different combinations of the CTL killing rate parameter (*k*_*e*_) and the parameter controlling the sensitivity of cell-cycle arrest to IFNG (*k*_*i*_). (E and F) Predictions for tumor growth rate (E) and ratio of tumor cells in S-G_2_-M:G_1_ states (F) for the combination of parameters with the highest likelihood (solid red line). In addition, simulations are shown with either the best-fitting k_i_ parameter and k_e_ = 0 (blue long dashed line) or the best-fitting k_e_ parameter and k_i_ = 0 (green short dashed line). Black points and error bars in (E and F) represent (respectively) mean ± standard deviation of experimental measurements. (G) Fractional reduction in transition rate from G_1_ to S-G_2_-M phase with varying *Ifng* expression levels, for the best-fitting parameter value k_i_ = 8.1 IFN^−1^ mm^3^. Dashed lines in C and G highlight the predicted reduction in transition rate from G_1_ to S-G_2_-M phase for the mean *Ifng* expression levels measured on days 3 (cyan), 5 (magenta), and 7 (yellow).

Due to the early reduction in IFNG signaling, it is unclear whether IFNG can be entirely responsible for the G_1_-phase tumor cell-cycle arrest, which followed highly similar dynamics to the CTLs. To test the compatibility of the *Ifng* transcription data with the dynamics of the CTLs and the tumor cell-cycle dynamics, we developed an ODE model. Our ODE model describing the interactions between CTLs and the tumor (Figure 4A) features an explicit description of the cell cycle of tumor cells, in which they cycle from G_1_ phase into S-G_2_-M phases at rate k_gs_ and then back into G_1_ phase at rate k_sg_. The model also features CTLs, which kill tumor cells at rate k_e_ and produce IFNG, which precludes tumor cells from transferring from G1 phase to S phase. The sensitivity of tumor cell-cycle arrest to IFNG is determined by the parameter k_i_. To test the contribution of the two CTL effector functions to tumor control (i.e., killing and antiproliferative effect), we linearly interpolated between the experimental data for the number of CTLs (Figure 4B) and for *Ifng* expression (Figure 4C) and used these interpolations directly as inputs to our model. Subsequently, we tested different combinations of the parameters k_e_ and k_i_ (Figure 4D) to find the best fit to the tumor growth rate (Figure 4E, red line) and the S-G_2_-M:G_1_ ratios (Figure 4F, red line) determined from the experimental data. Our best-fitting parameter set (Figure 4D; marked with black circle) had a value of k_e_ = 0.9 (CTL^−1^ day^−1^) although other values for k_e_ in the range 0–3 (CTL^−1^ day^−1^) led to relatively low errors, consistent with killing rates we have previously estimated for CTLs against B16F10 melanoma tumors.^19^ The best-fitting value for the antiproliferative effect (k_i_ = 8.1 IFN^−1^ mm^3^) led to sharp reductions in the transition rate of tumor cells out of the G_1_ phase for the *Ifng* expression levels found in our data (Figure 4G). At the peak of *Ifng* expression on day 3, the transition rate from G_1_ to S-G_2_-M phases (k_gs_) was reduced to 7% of its original value, and even at the lower *Ifng* expression levels measured on other days, k_gs_ was significantly reduced (Figures 4C and 4G; dashed lines). Thus, our best-fitting parameters implied that cycling tumor cells are sensitive to IFNG even at low expression levels.

When we took the best-fitting parameters and disabled killing by setting k_e_ = 0 (Figure 4D; marked with black square), most of the tumor growth reduction was preserved (Figure 4E, blue dashed line). In contrast, taking our best-fitting parameters and disabling the antiproliferative effect of IFNG (Figure 4D; marked with triangle) resulted in only a very small reduction in the net growth rate of the tumors (Figure 4E, green dashed line). These results show that for the best-fitting parameter combination, the antiproliferative effect rather than CTL cytotoxicity played a dominant role in tumor control. We also tested whether only cytotoxicity, or only an antiproliferative effect, was compatible with the data by fitting a reduced model with k_e_ or k_i_ set equal to zero (Figure S2). The Akaike information criterion (AIC) of the full model was 8.0; for the model without killing, the AIC was 6.7, and for the model without antiproliferative effect, the AIC was 131.3. Overall, these results support our previous analysis showing that an antiproliferative effect of IFNG is more important than CTL cytotoxicity to control B16F10 tumors.^19^ Moreover, these results show that the dynamics of IFNG are compatible with the dynamics of the tumor cell-cycle arrest, despite the apparently short duration of IFNG production.

### CTL exhaustion quantitatively explains *Ifng* transcription dynamics

**Figure 5:**
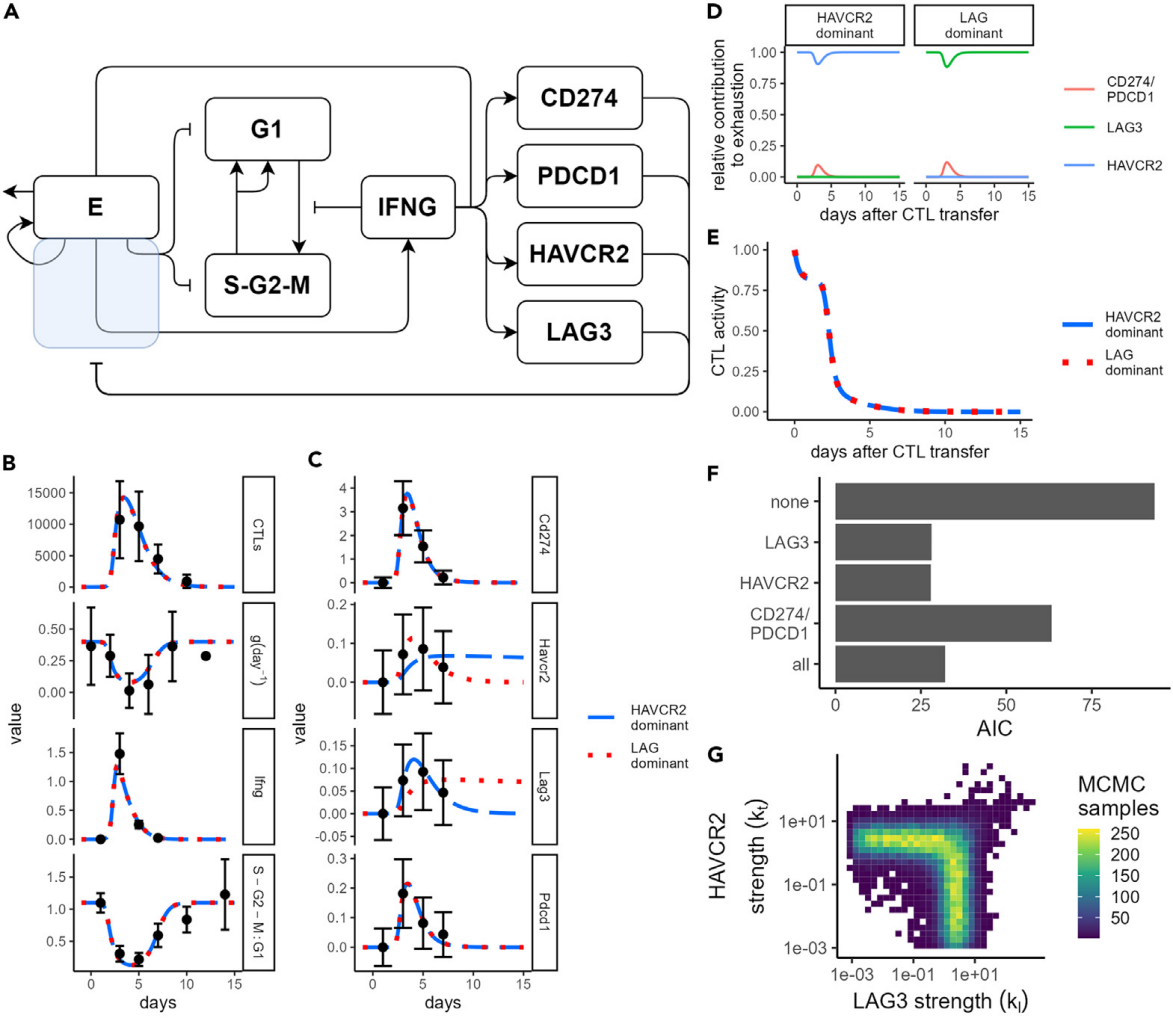
Early cessation of IFNG is quantitatively compatible with the development of LAG3- or HAVCR2-mediated CTL exhaustion (A) Schematic for ODE model of CTLs versus B16F10 melanoma. Pointed arrows represent a positive effect (i.e., transfer, production, recruitment), while flat-headed arrows represent an inhibiting effect. CTL functions that are reduced due to CTL exhaustion are represented by lines passing through the translucent blue box. (B) Model fits to CTL density (top row), volumetric tumor growth (second row), *Ifng* mRNA expression (third row), or ratio of S-G_2_-M:G_1_ nuclei (bottom row). (C) Model fit to ICs for each considered combination of ICs used for fitting the model. Different ICs are shown in each row as indicated by facet label. Colored lines in B-C represent model output from either LAG-dominant or HAVCR2-dominant models as indicated. Black points and error bars in (B and C) represent (respectively) mean ± standard deviation of experimental measurements. (D) Relative contribution of different ICs to CTL exhaustion level, shown for LAG-dominant and HAVCR2-dominant parameter estimates. Different colored lines are the contribution for each term in Equation 11 normalized to sum to one (see STAR Methods), i.e., LAG3: $k_{l}L/E$; HAVCR2: $k_{t}H/E$; PDCD1/CD274: $k_{p}\left(P/E\right)\left(P_{L}V/V_{0}\right)$. (E) CTL activity level α (see STAR Methods), shown for LAG-dominant and HAVCR2-dominant parameter estimates. (F) Akaike information criterion for models fit with no inhibitors, all inhibitors, or individual inhibitors as indicated. (G) Estimated joint distribution of parameters k_l_ and k_t_, controlling the inhibitory strength of LAG3 and HAVCR2, respectively.

In order to explain the dynamics of CTLs and IFNG and to quantify the importance of different immune checkpoints in these dynamics, we extended our ODE model (Figure 5A). In this extended model, CTLs infiltrate the tumor at a basal rate s_0_, expand within the tumor at rate s_e_, and die with rate d_e_. In addition to their killing of tumor cells, CTLs inside the tumor produce IFNG. Finally our model includes the immune checkpoints LAG3, HAVCR2, PDCD1, and its ligand CD274, which decrease the activity of CTLs (see STAR Methods). We fit this ODE model simultaneously to all the experimental data discussed in Figures 1, 2, and 3 (see STAR Methods; best-fitting parameter sets provided in Table 1), resulting in a model, which nicely explained the observed dynamics (Figures 5B and 5C). Among the best-fitting parameter sets, we observed a tendency for either HAVCR2 or LAG3 to make the most significant contribution toward CTL exhaustion (Figure 5D). The dominant immune checkpoint tended to decay more slowly in the model fit (Figure 5C), but regardless of which inhibitor was dominant, CTL dynamics and tumor growth (Figure 5B) as well as total CTL activity (Figure 5E) were similar. Importantly, without using any checkpoints, we could not obtain a good fit to any of the experimental measurements (Figure S3 green lines), demonstrating that a regulatory mechanism like T cell exhaustion is required to explain the T cell anti-tumour response.

**Table 1:** Description of model parameters, with best-fitting parameter values

symbol	Best (LAG3)	Best (HAVCR2)	Best (CD274/PDCD1)	Best (all - LAG dom.)	Best (all HAVCR2 dom.)	Best (all)	Units	Description
s_0_	0.001	0.001	0.001	0.001	0.001	0.001 [-. 0.01]	(mm^−3^ day^−1^)	Infiltration rate of CTLs into tumor
s_e_	8.70	8.67	7.32	8.36	8.41	8.41 [6.5,23.7]	(day^−1^)	Expansion rate of CTL population within tumor
d_e_	0.77	0.77	0.59	0.80	0.80	0.80 [0.47, 0.94]	(day^−1^)	Death rate of CTLs inside tumor
d_i_	3.21	3.28	0.67	3.44	3.61	3.61 [0.86,37.3]	(day^−1^)	Rate at which IFNG disappears from the system.
k_gs_	1.64						(day^−1^)	Basal tumor cell transition rate from G_1_ to S-G_2_-M cell-cycle phases
k_sg_	0.66						(day^−1^)	Basal tumor cell transition rate from S-G_2_-M to G_1_ cell-cycle phase
k_i_	4.28	4.33	21.66	4.20	4.40	4.40 [0.91, 127.8]	(IFN^−1^ mm^3^)	Determines the concentration of IFNG required to prevent transfer of tumor cells from G_1_ to S-G_2_-M cell-cycle phases. k_i_^−1^ is the concentration of IFNG required to reduce the transition rate by 50%.
k_e_	20	20	0.33	100	100	100	(CTL^−1^ day^−1^)	Rate at which CTLs kill tumor cells
k_A_	1						(IFN^−1^ mm^3^)	Relative contribution of IFNG to immune checkpoint expression (fixed before fitting)
k_ex_	1						CTL^−1^	Determines level of immune checkpoint expression required to decrease CTL function (fixed before fitting)
k_l_	1.51	0	0	1.29	0.001	0.001 [-, 13.9]	(LAG^−1^)	Contribution of LAG3 toward CTL exhaustion
k_t_	0	1.51	0	0.001	1.37	1.37 [-, 13.3]	(TIM^−1^)	Contribution of HAVCR2 toward CTL exhaustion
k_p_	0	0	0.01	0.001	0.001	0.001 [-, 1.76]	(PD-1^−1^ PD-L1^−1^ mm^3^)	Contribution of PDCD1/CD274toward CTL exhaustion
d_l_	0.01	0.64	0.42	0.01	0.65	0.65 [-, -]	(day^−1^)	Disappearance rate of LAG3 inside the system
d_t_	0.71	0.01	0.017	0.71	0.01	0.01 [-, -]	(day^−1^)	Disappearance rate of HAVCR2 inside the system
d_p_	2.27	2.36	0.023	2.08	2.33	2.33 [-, -]	(day^−1^)	Disappearance rate of PDCD1 inside the system
d_pl_	2.32	2.41	0.015	2.29	2.48	2.48 [-, -]	(day^−1^)	Disappearance rate of CD274 inside the system

Columns 2–4 give values for immune checkpoints fit individually, columns 5–6 give values for either LAG-dominant or HAVCR2-dominant fits with all immune checkpoints included, and column 7 gives values for the best full model overall with all immune checkpoints included 1–99% credible intervals for parameter estimates for the best model are given in the format [1% CI, 99% CI], with a dash (−) indicating a particular bound could not be determined. Columns 8–9 provide parameter units and descriptions, respectively.

Since none of our best-fitting parameter sets predicted a significant role for PDCD1-CD274 inhibition in the *in vivo* experimental setting with B16F10 tumors, we asked how important the small early contribution of PDCD1-CD274 to CTL exhaustion was for the model dynamics. To address this, we disabled each inhibitor in turn. In the LAG3-dominant model (Figure S4, red lines), only disabling LAG3 had a significant effect on model dynamics, but disabling PDCD1/CD274 or HAVCR2 had negligible effect. Similar results were obtained for the HAVCR2-dominant model (Figure S4, blue lines) in which only disabling HAVCR2 disrupted model dynamics, but disabling PDCD1/CD274 or LAG3 had no effect. These results suggest that inhibition by either LAG3 or HAVCR2 is required to explain the experimental data, but PDCD1/CD274 inhibition is dispensable. However, it could be that our stochastic optimization procedure simply did not find any parameter sets where PDCD1/CD274 inhibition was important, so we also tried fitting our model with each immune checkpoint separately. Including only PDCD1-CD274 as an inhibitor (purple lines; Figure S3) resulted in a poor fit to the number of CTLs counted inside the tumor, as well as the dynamics of PDCD1 and CD274 themselves. In contrast, with LAG3 (Figure S3, blue dashed lines) or HAVCR2 (Figure S3, red dotted lines) as sole inhibitors, we achieved an equally good fit as with the full model including all checkpoints together (Figure S3, black lines). Application of the AIC to our fits confirmed that having PDCD1/CD274 as the only inhibitors led to significantly worse performance than the other tested models (Figure 5F), while the models with only HAVCR2 or only LAG3 had a slightly improved score than the full model because of the need to estimate fewer parameters. Note that these model variants gave similar predictions to the simplified model, where CTLs and IFNG were used as inputs to the model, in terms of the relative importance of cytotoxic effects versus antiproliferative effects (Figure S5).

To further verify the relative importance of each checkpoint, we utilized a variant of the Metropolis Hastings algorithm to assess the practical identifiability of our estimated parameters. Four of our estimated parameters (d_e_, d_i_, k_i_, s_e_) were fully identifiable (Figure S6). To investigate why the parameters controlling the disappearance rate of the immune checkpoints were not identifiable, we took our best-fitting (HAVCR2-dominant) parameter set and swept the values of d_l_, d_t_, d_p_, and d_pl_ in a large range while holding all other parameter values fixed (Figure S7A). Even extremely high or low values of d_l_, d_p_, or d_pl_ led to relatively moderate decreases in log likelihood, whereas the log likelihood decreased more strongly for large values of d_t_. This occurs because, in this setting, only HAVCR2 contributes significantly to exhaustion, so modifying d_l_, d_p_, or d_pl_ has hardly any effect on model dynamics except for those of the inhibitor in question. Thus, the uncertainty in the currently used immune checkpoint data is too great to accurately estimate the values of the immune checkpoint disappearance rates. Moreover, parameters controlling the contribution of each immune checkpoint toward CTL exhaustion (k_p_, k_t_, k_l_) were not fully identifiable because the minimum credible value for all of these parameters was unbounded on a logarithmic scale. Importantly, however, k_p_ (contribution of PDCD1/CD274 toward CTL exhaustion) was predicted to be close to zero (Figure S6), while either k_t_ or k_l_ (contributions of respectively HAVCR2 and LAG3 toward CTL exhaustion) had to have a high value (Figure 5G), which generally co-occurred with a low value for the disappearance rate for the respective checkpoint (Figures S7B and S7C). Simulations with parameter sets drawn from the estimated posterior distributions described the data quite well but revealed 2–3 main trajectories suggesting the possibility of multiple local optima (Figures S7D and S7E). Coloring by parameter values revealed that two trajectories were enriched for either low or high values of the parameter d_p_ (Figure S8A). The other two trajectories that stood out were enriched for either low or high values of d_l_ (Figure S8B) and d_t_ (Figure S8C) reflecting the previously identified HAVCR2-dominant and LAG-dominant fits. Otherwise, there was little correlation between fitted parameters (Figure S9).

Overall, these results show that the brief window of IFNG production is quantitatively consistent with development of an exhausted state among CTLs and that expression of the immune checkpoint molecules LAG3 and HAVCR2 correlates best with the development of this exhausted state. Our analysis suggests that, of the three immune checkpoints considered, PDCD1/CD274 is the least important determinant of the exhausted CTL state; however, our model remains compatible with PDCD1/CD274 playing a role in CTL exhaustion at early time points after CTL infiltration of the tumor.

## Discussion

In a previous study on which our work was built, Matsushita et al. found that tumor control of B16F10 melanoma by CTLs was mediated by a combination of cytotoxic and cytostatic effects, with cytostatic effects being due to IFNG-mediated cell-cycle arrest of the melanoma cells.^15^ However, the progression of cytostatic and antiproliferative effects over time was not explicitly explored. Here, we analyzed image data, tumor volume measurements, and transcriptomics data from the study by Matsushita et al.,^15^ using data acquired at multiple time points after CTL transfer. We found that the presence of CTLs inside the tumor strongly correlated with tumor cell-cycle arrest, as well as with the inhibition of volumetric tumor growth. However, IFNG signaling within tumors followed early dynamics, with CTLs primarily producing IFNG early after arrival in the tumors. Since IFNG loss preceded the recovery of tumor cell proliferation, it was unclear whether IFNG signaling could completely account for the observed tumor cell-cycle arrest and what role T cell exhaustion had in these processes. Therefore, we developed an ODE model to describe tumor growth, CTL infiltration, CTL production of IFNG and subsequent interference with cell-cycle progression, and also tumor cell killing by CTLs. Using this model we could describe all the experimental data, which led us to conclude that IFNG-mediated tumor cell-cycle arrest, together with killing of tumor cells by CTLs, was sufficient mechanism to account for the experimental data. We also used our models to compare the contribution of CTL-mediated cytotoxic or cytostatic effects toward tumor control. Our models predicted only a minor contribution of CTL killing toward tumor control compared to the IFNG-mediated cell-cycle arrest, consistent with our prior findings.^19^

As part of our study, we developed a model describing the dynamics and effector functions of tumor-infiltrating CTLs. Based on mRNA expression data, *Ifng* transcription peaked on day 3, had fallen sharply by day 5, and was virtually zero on day 7. This was in contrast to the number of CTLs, which remained present in similar numbers on days 3 and 5 and were still observable in reasonable numbers at late time points. Therefore, our model included the development of CTL exhaustion in order to account for this loss in ability to produce IFNG. Another alternative, which might explain reduced CTL function, is a reduction in stimulation via the T cell receptor, which could also account for the decreasing PDCD1 expression on the CTLs since PDCD1 is normally expressed during CTL activation.^28^ However, we consider this unlikely since the tumors remained large during the period of the experiments, meaning that CTLs should remain in contact with tumor cells and thus remain stimulated. CTL exhaustion is identified by a progressive increase in the number and diversity of inhibitory receptors expressed by CTLs.^25^^,^^29^^,^^30^ We identified four well-known inhibitory molecules among the available transcriptomics data: LAG3, HAVCR2, PDCD1, and the PDCD1 ligand CD274. With our model we were able to obtain good fits if the exhausted state was correlated with HAVCR2 or LAG3 but not with PDCD1/CD274, which was due to the early peak of *Pdcd1* and *Cd274* transcription that was already well in decline on day 5 while CTL numbers in the tumor remained high. This early peak was not compatible with the idea that CTLs were becoming gradually more exhausted over time. On the other hand, *Lag3* and *Havcr2* increased relative to the CTLs over time and therefore correlated most with the loss of *Ifng* transcription. Consistent with our model prediction, LAG3 and HAVCR2 have been previously shown to have high correlation with a dysfunctional “exhausted” phenotype in CD8^+^ CTLs in melanoma.^26^ Our model was not compatible with the dynamics of PDCD1/CD274 as sole correlates of the exhausted state, which appears at first sight to contradict reports indicating that PDCD1/CD274 signaling is relevant for immunosuppression in melanoma,^31^^,^^32^ although our result agrees with others showing that B16F10 in particular may be resistant to PDCD1 antagonist monotherapy.^33^^,^^34^ Since our model was compatible with PDCD1/CD274 signaling making a partial contribution to CTL exhaustion at early time points, it may be that CD274/PDCD1 plays only an initial role in immune suppression and that this role is taken over later by other checkpoints. This is consistent with findings that blockade of LAG3 as well as PDCD1 receptors is required to prevent relapse in melanoma^31^ and with recent clinical results demonstrating that co-administration of PDCD1 and LAG3 inhibitors improves survival compared to administration of PDCD1 inhibitors alone.^35^

One caveat for the data employed to fit our model is that only one probe was available per checkpoint. Therefore future experiments should confirm the dynamics of the expression of these immune checkpoint molecules and further investigate their contribution to T cell exhaustion. Another potential criticism of our approach is that it may be unfair to consider LAG3 and HAVCR2 separately from PDCD1/CD274-mediated immunosuppression since PDCD1 upregulation usually precedes LAG3 and HAVCR2 upregulation following T cell activation.^25^ Indeed, our model is phenomenological with respect to checkpoint dynamics, so any causal relationships, which may exist between the expression of different checkpoints, are not taken into account because additional data would be required to describe such causal interactions. Therefore, the immune checkpoint component of our model should be viewed as a means to explore hypothetical scenarios where only single checkpoints have a role in T cell exhaustion.

Our model implies that the reduced activity of CTLs and in particular the apparent reduction in IFNG, which preceded the disappearance of CTLs in the tumor by several days, could be explained by the development of an exhausted phenotype in the tumor-infiltrating CTLs. Moreover, in our model IFNG played an important role in driving this exhausted phenotype. For exhaustion related to the CD274/PDCD1 axis, this is clearly justified because IFNG can induce upregulation of CD274 on tumor cells.^13^ Moreover, IFNG induces increased antigen presentation on tumor cells,^12^ which should lead to increased stimulation of CTLs via their T cell receptors. This could explain the contribution of IFNG toward upregulation of the other immune checkpoints included in our model, which are more commonly associated with excessive and prolonged exposure to antigen.^30^ In order to further study the dynamics of the CTL population in the tumor, it would be useful to perform a second transfer of CTLs, which may help elucidate the extent to which the mechanisms of decline in CTL function are due to transferred CTLs becoming exhausted (and therefore a second transfer of “fresh” CTLs should result in similar anti-tumour effects) or are due to resistive mechanisms deployed by the tumor (in which case a second transfer of CTLs would be expected to provide only limited benefit).

In our analysis, we estimated several parameters, and these can be compared to values in the literature. A wide range of estimates (0.02–1 day^−1^) for the death rate of CTLs have been reported,^20^^,^^36^^,^^37^^,^^38^ which are compatible with our own estimate of 0.8 days^−1^. We also estimated that the infiltration rate of CTLs into the tumor was very low, with our estimate hitting the lower parameter bound of s_0_ = 0.001 CTLs mm^−3^ day^−1^. The lower bound of s_o_ was set on the basis that tumors were around 100 mm^3^ while CTLs were infiltrating, and one could interpret the value s_0_ = 0.001 CTLs mm^−3^ day^−1^ as a 10% chance of a single CTL infiltrating a tumor of size 100 mm^3^ per day. This value seems implausibly low, thus our modeling of CTL infiltration into the tumor as a constant rate process may be an oversimplification. Since s_0_ was low, the large numbers of CTLs in the tumor were instead explained by a significant contribution from CTL expansion inside the tumors (estimated as the high value of s_e_ = 8-9 days^−1^). CTLs have been reported to divide up to five times per day *in vivo*,^39^ which is in a similar range albeit somewhat lower than the 8–9 divisions per day we estimated. While the variability in tumor growth rates estimated for individual untreated mice (Figure S1) does increase uncertainty regarding estimates of other parameters, the early volume growth rates in the presence of CTLs matched the population growth rates in the absence of CTLs quite well, so we expect the impact of this variability to be low. In conclusion, there remains some uncertainty surrounding our parameter estimates for CTL infiltration and intratumoral expansion, underscoring the need for future experimental studies to measure the rates of both processes following adoptive CTL transfer. Importantly, we do not expect these uncertainties to affect our main conclusions regarding CTL killing and antiproliferative effects or the importance of various checkpoints. This is because those conclusions require the model to accurately describe the number of CTLs inside the tumors over time, which was the case.

In our analysis, we used mRNA expression as a substitute for protein expression. Previous studies report that mRNA levels are substantially predictive of protein expression levels.^40^^,^^41^ Moreover, although some delay should be expected between mRNA expression and protein expression, this delay has been estimated to last for only a few hours^41^ and thus should not have a significant impact on our data, which consist of measurements made across several days. For the *in vivo* setting we studied, the rapid decline in the S-G_2_-M:G_1_ ratio after transfer of CTLs indeed suggests that protein expression rapidly follows mRNA expression. Conversely, the S-G_2_-M:G_1_ ratio does not appear to recover immediately upon downregulation of *Ifng* mRNA. One explanation could be that the effect of IFNG lasts longer than the protein due to downstream signaling. Another is that tumor cells are very sensitive to low levels of IFNG, therefore the effect could persist even after IFNG synthesis has substantially declined. The latter explanation seems to be in line with a study, which found that bystander sensing of IFNG could occur at distances of over 40 cell lengths,^13^ implying high sensitivity of tumor cells to this cytokine.

In summary, we have presented a mathematical model that can successfully predict inhibition of tumor growth following adoptive T cell transfer. We used this model to quantify the contribution of IFNG and cytotoxicity to the anti-tumor activity of CTLs, which led to the conclusion that IFNG contributes most to tumor growth blockade by CTLs. Our model also includes anti-tumorigenic (antiproliferative, enhancing recruitment of CTLs) and pro-tumorigenic (driver of CTL exhaustion) effects of IFNG. The presence of opposing effects of IFNG has led to descriptions of an “IFNG paradox”.^12^ Our model, by including these different effects associated with IFNG, can serve as a quantitative baseline to be augmented in future and may help guide further experimental work.

### Limitations of the study

Our study was limited by a lack of direct data concerning several important aspects of the CTL dynamics within the tumor. First, we had no direct data on the killing rate (k_e_) of the CTLs inside the tumor, so this parameter was allowed to vary freely during the fitting process. Depending on which immune checkpoint molecules were included in the fitting process, we recovered a range of different values for k_e_, although these parameters were all plausible and comparable with other values for the killing rate of tumor cells by CTLs *in vivo* reported elsewhere,^42^ including that for attack of B16F10 cells.^19^ Interestingly, the higher killing rate estimated using the model with immune checkpoints implies that the low predicted impact of killing on the tumors in these experiments might have been due to checkpoint-mediated suppression of killing. This shows that it is difficult to obtain proper “intrinsic” killing rate estimates without direct measurement. Importantly, however, the choice of immune checkpoint molecules and the resultant values of k_e_ did not impact our conclusion that IFNG-mediated cell-cycle arrest was the main determinant of tumor control. A second limitation surrounds our model of CTL exhaustion inside the tumors. Two specific questions we could not address due to the whole-tumour microarray data we used were: 1) which cells were expressing inhibitory molecules and 2) whether our results would have been different had we included “missing” relevant molecules from the transcriptomics data, e.g., the immune checkpoint CTLA-4. Unbiased gene expression data generated at the single-cell level, using single-cell RNA sequencing techniques, would therefore be interesting to incorporate into similar modeling strategies in future. A third limitation is the possible presence of other tumor-infiltrating cells, such as MDSCs which are recruited to B16F10 tumors after adoptive transfer of CTLs and exert suppressive effects on the tumor-infiltrating CTLs.^43^ The frequency of various immune cell types can be inferred from either single cell or bulk transcriptomic data using computational methods,^44^ and it would be interesting to extend our modeling approach to include other relevant immune cells using such methods in future. Finally, our estimates of the numbers of tumor cells and CTLs inside the tumor are based on thin 2D tumor sections. Since CTLs are smaller than tumor cells, they are more likely to remain undetected within the thin tissue slices, possibly resulting in an underestimate of the CTL to tumor cell ratio. It should be noted though that with image-based approaches typically more T cells are detected compared to approaches involving cell isolation procedures.^45^ Nevertheless, using images based on 3D image stacks to determine the densities of cells inside tumors would likely result in more accurate estimates in future.

## STAR★Methods

### Key resources table

**Table:** 

REAGENT or RESOURCE	SOURCE	IDENTIFIER
**Deposited data**		

Gene expression data	Matsushita et al.^15^	Gene Expression Omnibus (GEO) database (series GSE57304; samples GSM1379331– GSM1379344)
Raw and analyzed data	This paper	https://doi.org/10.5281/zenodo.7779313

**Software and algorithms**		

Ilastik	Berg et al.^22^	https://www.ilastik.org/
Model and analysis source code	This paper	https://doi.org/10.5281/zenodo.7779313

### Resource availability

#### Lead contact

Further information and requests for resources should be directed to and will be fulfilled by the lead contact, Joost Beltman (j.b.beltman@lacdr.leidenuniv.nl).

#### Materials availability

This study did not generate new unique reagents.

### Method details

#### Data summary

For the development of the mathematical model, data from the paper by Matsushita et al.^15^ were used. In brief, the experimental protocol in that previous study involved inoculation with 10^7^ B16F10 melanoma cells into C57BL/6 mice, followed 9 days later by adoptive transfer of 10^6^ activated pmel-1 transgenic T cells recognising the gp100 peptide (note that throughout the current study, the day of CTL transfer is designated “day 0”). The data included measurements of tumor volume from experiments in mice either with or without subsequent adoptive transfer of CTLs. From the same experiments we also used fluorescence microscope images of cryosections of B16F10 tumors expressing the FUCCI cell cycle sensor, taken on days 1, 3, 5, 7, 10 & 14 following CTL transfer. Finally, the dataset included microarray RNA expression data from B16F10 tumors at days 1, 3, 5 & 7 after CTL transfer. In the experimental data, multiple tumor volume measurements were obtained from the same mouse at different timepoints. However, for the imaging and RNA expression data, each time point represents data from a different mouse since sacrifice of the mouse was required to obtain the measurements.

#### Image analysis

Automated estimates of the number of G_1_ or S-G_2_-M phase nuclei were produced using the ilastik (version 1.1.3) cell density estimation tool. Training and classification was performed using merged (RGB) images. For training the classifier we selected subregions (100-200 μm^2^) from the larger (750 × 550μm) cryosections. One subregion was selected from each available time point to ensure a representative training set. Pipelines for different nuclei (G_1_ or S-G_2_-M) were trained separately. Training was performed by manually labeling training images until the classifier estimated numbers of cells achieved a satisfactory match with manual counts for the same data.

#### Analysis of gene expression data

Microarray data were downloaded from the Gene Expression Omnibus (GEO) database (series GSE57304; samples GSM1379331– GSM1379344). These data correspond to the same set of experiments as the image and tumor volume progression data we have used, and the methodology for acquisition of these data has been described previously.^15^ Briefly, tumor tissues from mice were harvested on different days (1, 3, 5, 7) after CTL transfer, or on the same days in the untreated (without CTLs) condition. Each sample contained 500 ng of pooled RNA from 3 to 4 different tumors, and microarray analysis was performed with 45,018 probes to quantify expression levels of the targeted genes. We performed similar data processing steps to the original publication: probes were discarded when their gIsWellAboveBG flag was zero at all samples, and we normalised different samples at the 75th percentile.

#### Basic ODE model

We developed an ODE model to describe the intratumoral activities of the transferred CTLs. The basic model of tumor growth (in the absence of CTLs) considers two possible states for alive tumor cells: they can be either in the G_1_ phase of the cell cycle (denoted in the equation as G), or else in the S, G_2_, or M phase (together denoted S in the model equations). The reason for choosing these states as explicit model variables was because the FUCCI cell cycle reporter used in the experiments, which our model is based upon, could distinguish only between G_1_ or S-G_2_-M phases. Cells move from the G_1_ state into the S-G_2_-M state at rate k_gs_, and leave the S-G_2_-M state at rate k_sg_ (Equation 1):

(Equation 1) $$\frac{dS}{dt}=k_{gs}G-k_{sg}S\text{.}$$

The S-G_2_-M state concludes when a tumor cell undergoes mitosis. To include this increase in tumor cells in our model, we consider that for every cell which leaves the S-G_2_-M state, two cells enter the G_1_ state (Equation 2):

(Equation 2) $$\frac{dG}{dt}={-k}_{gs}G+2k_{sg}S\text{.}$$

The resulting tumors grow exponentially when the ratio of cells in G and S states is at its steady state value. When CTLs (*E*) are introduced into the tumors, our basal model of tumor growth (Equations 1 and 2) is modified to include two possible effects CTLs can have on the tumor. The first of these effects is direct killing of tumor cells, which occurs with a constant rate $k_{e}$ (per CTL). As we have done previously,^18^^,^^19^ we take the total killing activity of CTLs to be directly proportional to the number of CTLs inside the tumor, such that the total killing activity of the CTLs is given by ***α***k_e_E. Here, α is a scalar used to modify the effector functions of CTLs if their activity is reduced due to being exhausted (see (Equation 7), (Equation 8), (Equation 9), (Equation 10), (Equation 11), (Equation 12) below). Note that in general the total amount of CTL killing is expected to saturate with CTL and/or target cell density, and can be described with a double saturation (DS) function. However, in a situation where the target cell density greatly exceeds the CTL density, as is the case in our modeled scenarios, this DS function reduces to the here employed (simpler) killing term.^18^^,^^19^^,^^20^ We consider that killing is directed equally toward cells in G_1_ or S-G_2_-M phases, so that the fraction of tumor cells in either state (i.e. $G\;{\left(S+G\right)}^{-1}$ or $S\;{\left(S+G\right)}^{-1}$, respectively) determines the fraction of the total killing activity that each subset of tumor cells receives.

The second effect that CTLs can have on the tumor is an antiproliferative effect, mediated by IFNG, which results in an arrest of the cell cycle in the G_1_ phase. To include this effect in our model we reduce the transition of cells out of the G_1_ phase by scaling with the term ${\left(1+k_{i}I/V\right)}^{-1}$. Here, the variable I represents the total quantity of IFNG inside the tumor and V is the variable representing tumor volume. Thus, the term I/V represents the concentration of IFNG inside the tumors, and $k_{i}$ determines the concentration dependence of the IFNG dependent reduction in the rate at which tumor cells can leave the G_1_ phase. The equations to describe the evolution of the number of tumor cells in G_1_ or S-G_2_-M phases become:

(Equation 3) $$\frac{dS}{dt}=k_{gs}G\;{\left(1+k_{i}I/V\right)}^{-1}-k_{sg}S-{\alpha k}_{e}\;E\;S\;{\left(S+G\right)}^{-1}\text{,}$$

(Equation 4) $$\frac{dG}{dt}={-k}_{gs}G\;{\left(1+k_{i}I/V\right)}^{-1}+2k_{sg}\cdot S-{\alpha k}_{e}\;E\;G\;{\left(S+G\right)}^{-1}\text{.}$$

To test the compatibility of *Ifng* transcription dynamics with G_1_ tumor cell-cycle arrest Equations 3 and 4 were used directly. *I* and *E* were estimated by linearly interpolating between the mean of the experimental data at each available time point, and these linear interpolations were used as inputs to the model.

#### ODE model with CTL dynamics

To describe the dynamics of the CTL population and their production of IFNG, we extended our basic ODE model with further equations. We consider that after transfer, CTLs would begin to arrive in any given region of the tumor at a constant rate $s_{0}$. We take a constant rate of CTL arrival per unit volume of tumor, hence the rate at which CTLs can find the tumor scales with tumor volume. Moreover, we consider that CTLs expand within the tumor at rate $s_{e}$, and die at a constant rate, $d_{E}$. CTL expansion inside the tumor is also reduced according to the level of CTL exhaustion (α). Thus, CTL dynamics is described by the following equation:

(Equation 5) $$\frac{dE}{dt}=s_{0}V+{\alpha s}_{e}E-d_{E}E\text{.}$$

We consider CTLs to be the major source of IFNG inside the tumors, therefore IFNG production is proportional to the number of CTLs, but is reduced according to their level of exhaustion (α), and IFNG disappears from the system with a rate $d_{i}$:

(Equation 6) $$\frac{dI}{dt}=\alpha E-d_{i}I\text{.}$$

Finally, we include a mechanism whereby CTLs become exhausted inside the tumor. T cell exhaustion is characterised by a loss of effector functions along with a progressive increase in the amount and diversity of inhibitory receptors expressed by T cells.^25^^,^^29^^,^^30^ We used the well described PDCD1, CD274, LAG3 & HAVCR2 inhibitory molecules as indicators of exhausted T cells,^26^ which in our model appear with variable names P, P_L_, L, and H (respectively):

(Equation 7) $$\frac{dP}{dt}=E\left(1+k_{A}I/V\right)-d_{p}P\text{,}$$

(Equation 8) $$\frac{dP_{L}}{dt}=E\left(1+k_{A}I/V\right)-d_{pl}P_{L}\text{,}$$

(Equation 9) $$\frac{dL}{dt}=E\left(1+k_{A}I/V\right)-d_{l}L\text{,}$$

(Equation 10) $$\frac{dH}{dt}=E\left(1+k_{A}I/V\right)-d_{t}H\text{.}$$

We tested several model variants, one with no immune checkpoints, three in which we consider one checkpoint at a time, and one considering all checkpoints simultaneously. All inhibitory molecules follow similar dynamics, increasing in proportion to the number of CTLs inside the tumor and disappearing from the system with different rate constants d_p_,d_pl_,d_l_, and d_t_ (respectively). Production is increased proportional to the term $\left(1+k_{A}I/V\right)$, which allows for a contribution of IFNG to the exhausted state. Note that the IFNG induction of exhaustion may be direct or indirect, e.g. by increasing antigenicity of tumor cells and thereby increasing stimulation of T cells via the T cell receptor. In our model, we consider PDCD1, LAG3, and HAVCR2 as expressed on the membrane of CTLs, so the ratio of each of these checkpoint molecules to the number of CTLs determines the overall level of exhaustion of the CTLs in our model. CD274 is modeled differently, being a ligand for the PDCD1 receptor, and the concentration of CD274 in our model is multiplied together with the membrane density of PDCD1 expressed on CTLs to determine the contribution from PDCD1-CD274 signaling (see Equation 11 in ref. ^20^). To describe the joint effect of these inhibitory molecules on the CTLs, we consider a weighted sum *R*:

(Equation 11) $$R=k_{l}L/E+k_{t}H/E+k_{p}\left(P/E\right)\left(P_{L}/V\right)$$

*R* represents the total “exhaustedness” of the CTL population inside the tumor. The parameters k_l_, k_t_, and k_p_ represent the individual contribution of (respectively) LAG3, HAVCR2, and PDCD1/CD274 signaling toward the level of exhaustion of CTLs. In absence of detailed information about the impact of exhaustion level on CTL functions (killing, IFNG production, expansion), we take all these functions to be equally reduced with the level of CTL exhaustion:

(Equation 12) $$\alpha =1-{\left(1+\frac{k_{ex}}{R}\right)}^{-1}\text{.}$$

Here, $k_{ex}$ is the level of exhaustion at which all effector functions are half of their maximum value. Thus, Equation 12 scales the exhaustion level to a scaled term α which can range from 0 to 1 and is applied to the relevant rate constants in Equations 3, 4, 5, and 6.

### Quantification and statistical analysis

#### Parameter estimation

Parameter estimation for the two parameters in the basal tumor growth model was performed separately from the other parameters. We consider that the density of the tumor cells remained constant over time, so that volumetric tumor growth rate could be taken as a proxy for the expansion rate of the tumor cell population. We obtained an estimate for the untreated tumor growth rate (*g*) from fitting an exponential model of tumor growth to the volumetric growth data for the untreated tumors. Moreover, estimates of the ratio of tumor cells in the S-G_2_-M: G_1_ phase gave a second measurement allowing the two-parameter (k_gs_, k_sg_) basic model of tumor growth to be completely defined, considering that the ratio of S-G_2_-M: G_1_ phase tumor cells has reached a steady state (which is reasonable since we deal with data two weeks after tumor inoculation). Then, using the equation for exponential growth:

(Equation 13) $$\frac{d\left(S+G\right)}{dt}=g\;\left(S+G\right)\text{,}$$

where g is the tumor growth rate, one can substitute the left hand side of Equation 13 with $k_{sg}S$, noting that $k_{sg}S$ is the total rate of new tumor cell production obtained from summing Equations 1 and 2. Following the substitution, an expression for *g* can be found in terms of S and G:

(Equation 14) $$g=k_{sg}S\;/\;\left(S+G\right)\text{.}$$

Since our model results in an exponentially growing tumor with a constant ratio of cells in S:G states, explicit equations for the growth of the populations in each state can be written separately:

(Equation 15) $$S\left(t\right)=S_{ss}e^{gt}\text{,}$$

(Equation 16) $$G\left(t\right)=G_{ss}e^{gt}\text{,}$$

with the subscript (SS) indicating validity of these equations when the initial populations are at their steady state ratio. Following differentiation of each equation:

(Equation 17) $$\frac{dS}{dt}={gS}_{ss}e^{gt}\text{,}$$

(Equation 18) $$\frac{dG}{dt}={gG}_{ss}e^{gt}\text{,}$$

The resulting Equations 17 and 18 can be combined to remove the common terms ($ge^{gt}$). This leads to the following:

(Equation 19) $$\frac{1}{S}\frac{dS}{dt}=\frac{1}{G}\frac{dG}{dt}\text{,}$$

where we have omitted the subscript with the understanding that Equation 19 is valid only when the tumor is growing exponentially with the ratio S/G at a steady state value. Therefore, by substituting the expressions for $\frac{dG}{dt}$ and $\frac{dS}{dt}$ given in Equations 1 and 2, the Equations 14 and 19 can be rearranged to express the $k_{sg}$ and $k_{gs}$ parameters for the basal tumor growth model as a function of tumor growth rate (*g*) and the ratio of S-G_2_-M: G_1_ phase tumor cells at steady state:

(Equation 20) $$k_{sg}=g\;\left(1+S/G\right){\left(S/G\right)}^{-1}\text{,}$$

(Equation 21) $$k_{gs}=g\;\left(1+2S/G\right)$$

Before estimating the remaining model parameters, we used the SIAN tool to check whether our model was structurally identifiable.^46^ This revealed that the parameters k_A_ and k_ex_ were not identifiable, so we set both values equal to 1. The remaining model parameters which relate to the dynamics of the CTLs and their effects on the tumors were obtained together, by fitting to all available data over time simultaneously (i.e. the CTL counts; the S/G ratios; the volumetric growth data; the *Ifng* expression data; the immune checkpoint expression data). To determine the optimal parameters we maximised the log likelihood:

(Equation 22) $$\log\;L=-\frac{1}{2}\sum_{m}^{M}\sum_{j}^{J}\frac{{\left(y_{m}\left(t_{j}\right)-{\bar{y}}_{m,j}\right)}^{2}}{\sigma_{m,j}^{2}}.$$

Where $y_{m}\left(t_{j}\right)$ is the model estimate for observable m output at time point $t_{j}$, ${\bar{y}}_{m,j}$ and $\sigma_{m,j}^{2}$ are respectively the mean and standard deviation of the corresponding experimental measurements. For the immune checkpoints there was only one sample per time point corresponding to the CTL treated condition, precluding calculation of the standard deviation. Therefore we estimated the standard deviation of the checkpoints based on the standard deviation of a list of housekeeping genes^47^ (most stable mouse transcripts). A linear regression of the mean and standard deviation expression of the housekeeping genes in our data gave the relation: $\sigma =0.054+0.29\bar{y}$, with adjusted R-squared 0.986. This relation was used to estimate the standard deviation for each individual immune checkpoint. Growth rate measurements between days 10–14 had an outsized effect on our optimization due to their very low standard deviation originating from only two mice in a single experiment, thus were excluded from fitting.

For the basic model with I and E used as inputs, there were only two parameters (k_i_ and k_e_) to be estimated, therefore we tested all combinations of the parameters in the range 0–20 at intervals of 0.1 and selected the combination with the highest log likelihood. For the model including CTL dynamics we used a multi-start strategy with 20,000 initial parameter guesses selected using latin hypercube sampling, then optimised using the L-BFGS-B algorithm. We set relatively permissive bounds on the parameters as follows: all protein degradation parameters (d_i_, d_t_, d_l_, d_p_, d_pl_) between 0.01 and 100 days^−1^, corresponding to half-lives between 10 min and 70 days. Bounds of 0.01–100 days^−1^ were used for the killing rate of CTLs, and bounds of 0.01–10 days^−1^ were used for CTL death rate. All other parameters were bounded in the range 10^−3^ to 10^3^. Best-fitting parameter sets are provided in Table 1 in main text.

To approximate the posterior distribution of the fitted parameters, we used the Adaptive Metropolis Parallel Hierarchical Sampling algorithm.^48^ We ran 20 auxiliary chains alongside the main chain, using the best 21 parameter sets derived from the multi-start optimisation procedure for initialisation. Chains were run for 100,000 steps and the final 50,000 samples of the main chain were used for sampling.

## Data Availability

• Gene expression data analyzed in this study were obtained from the Gene Expression Omnibus (GEO) database (series GSE57304; samples GSM1379331– GSM1379344). All other data have been deposited on github and are publicly available as of the date of publication. DOIs are listed in the key resources table. • All original code has been deposited on github and is publicly available as of the date of publication. DOIs are listed in the key resources table. • Any additional information required to reanalyze the data reported in this paper is available from the lead contact upon request.
