# Supplementary material for: Mathematical modeling identifies LAG3 and HAVCR2 as biomarkers of T cell exhaustion in melanoma
Source: iScience. 2023 Apr 13;26(5):106666. doi: 10.1016/j.isci.2023.106666 (PMC10173735; doi:10.1016/j.isci.2023.106666)
Supplement: Document S1. Figures S1–S9 [file mmc1.pdf]

iScience, Volume *26*

Supplemental information

Mathematical modeling identifies LAG3 and HAVCR2 as biomarkers of T cell exhaustion in melanoma

Richard J. Beck, Sander Sloot, Hirokazu Matsushita, Kazuhiro Kakimi, and Joost B. Beltman

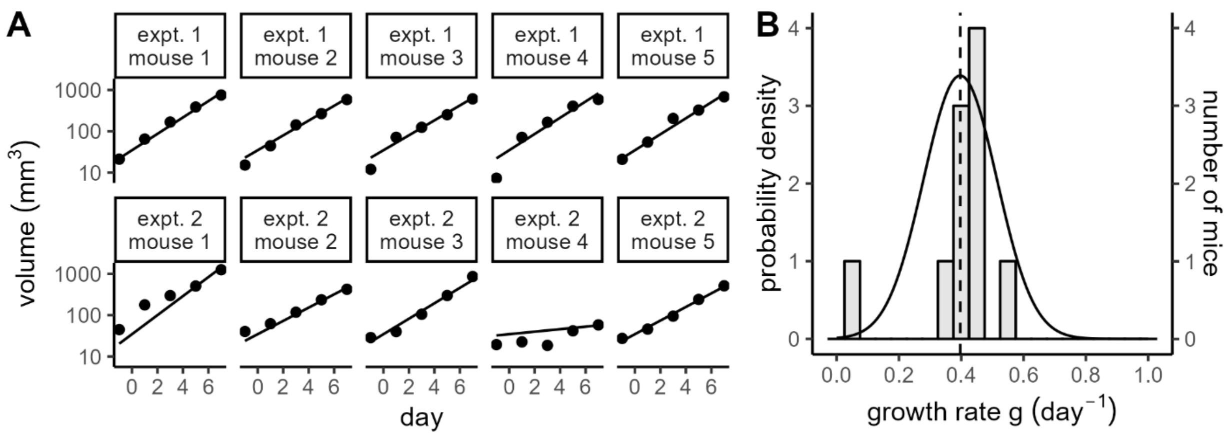

Figure S1. Mixed effects model of tumour growth in individual mice, related to Figure 2. A) Exponential growth model fit to individual mice. The initial tumour volume was treated as a fixed effect and a single value was estimated for all mice jointly. The tumour growth rate was treated as a normally distributed random effect and rates were estimated separately per mouse. B) The estimated probability density function (pdf) for tumour growth rate (line) is shown alongside the distribution of growth rates estimated per mouse (bars). Dashed vertical line indicates mean of estimated pdf.

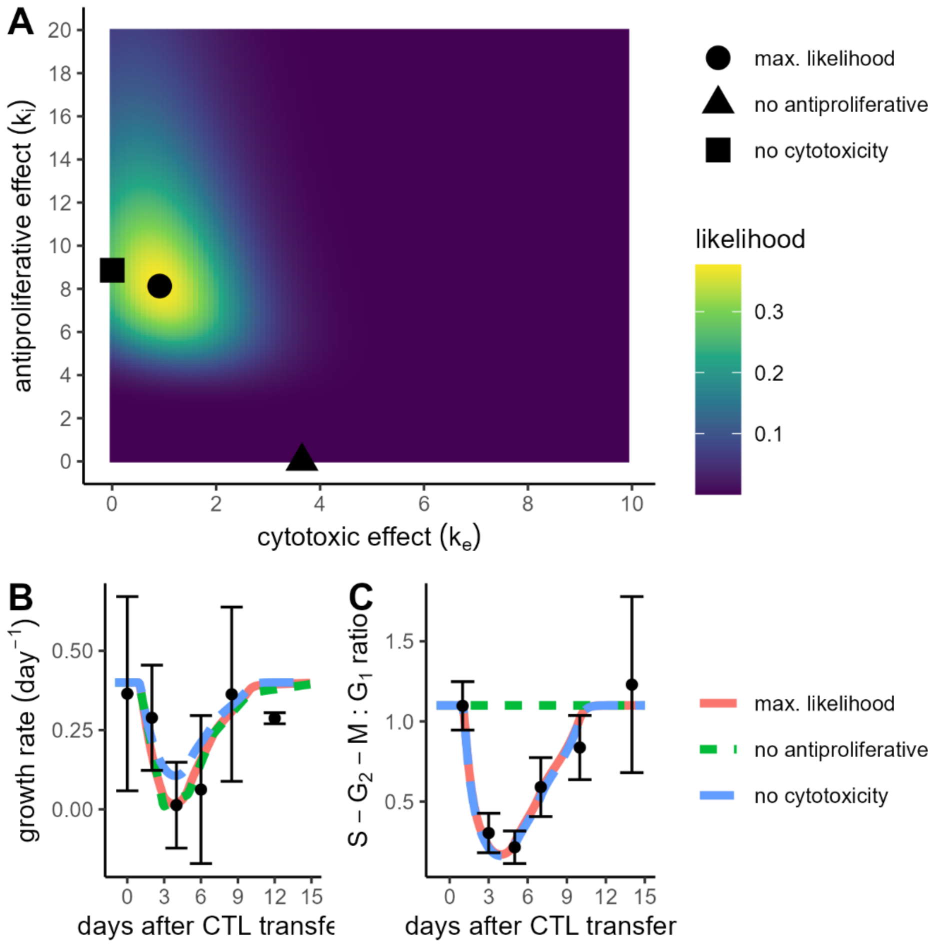

Figure S2. Compatibility of solely cytotoxicity or antiproliferative effects with tumour control and cell cycle arrest, related to Figure 4. A) Heatmap showing likelihood for different combinations of the CTL killing rate parameter (k_e) and the parameter controlling the sensitivity of cell-cycle arrest to IFNG (k_i). Points show maximum likelihood estimates for the model with both killing and antiproliferative effects included (circle), for the model with only killing (triangle), or the model with only antiproliferative effects (square). B-C) Predictions for tumour growth rate (B) and ratio of tumour cells in S-G_2-M : G_1 states (C) for the combination of parameters with the highest likelihood (solid red line). In addition, simulations are shown with either the best fitting model without cytotoxicity (blue long dashed line), or with the best fitting model without antiproliferative effects (green short dashed line). Black points and error bars in B-C represent (respectively) mean +/− standard deviation of experimental measurements.

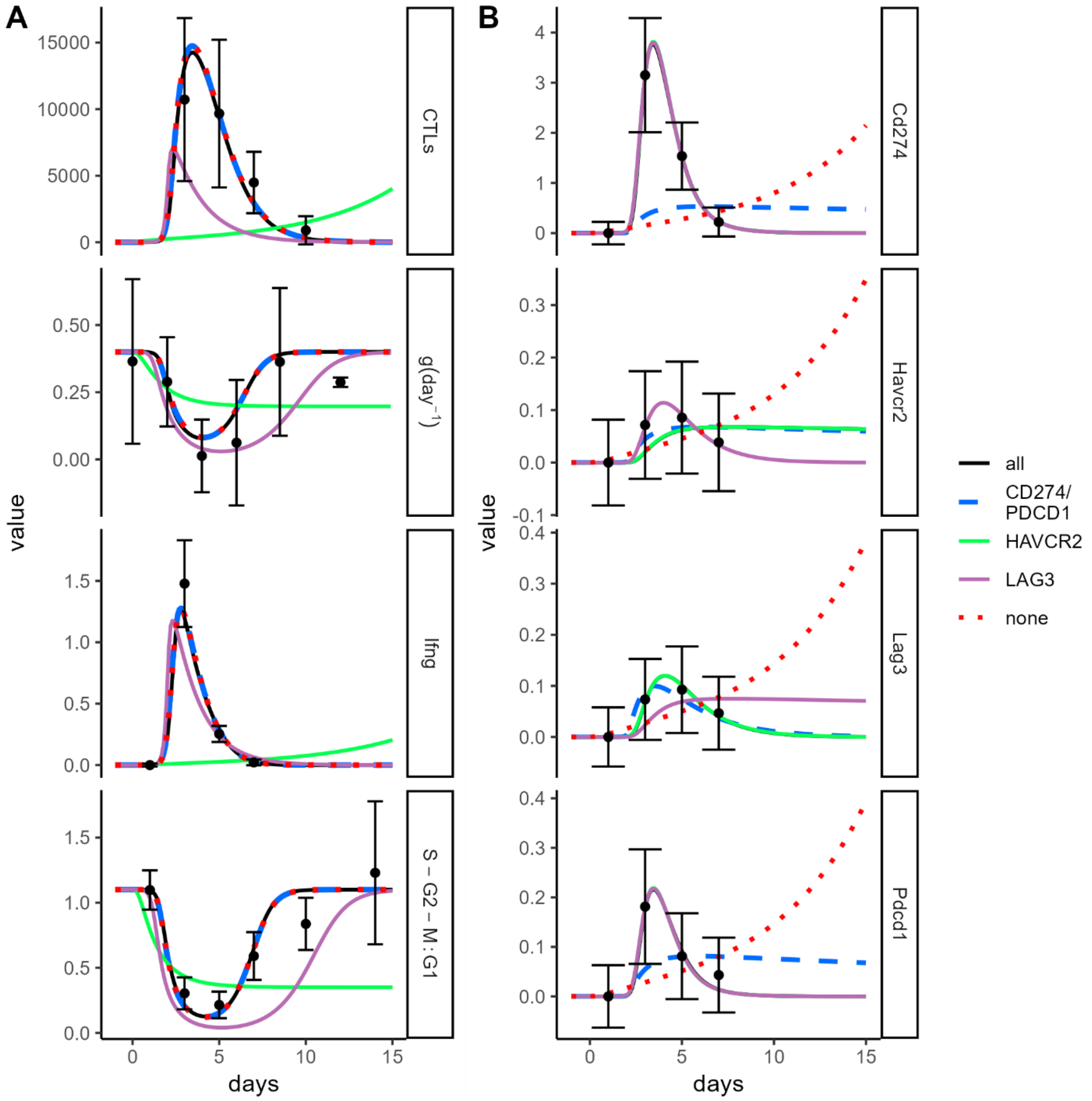

Figure S3. Parameter estimation results using subsets of checkpoints, related to Figure 5. A) Model predictions and experimental data for CTL density (top row), volumetric tumour growth (2nd row), *Ifng* mRNA expression (3rd row), or the ratio of S-G_2-M:G_1 nuclei (bottom row). B) Model predictions and experimental data for ICs, with different ICs shown in each row as indicated by facet labels. Results (A-B) are from fitting the model using no inhibitors, all 3 inhibitors, or a single inhibitor as indicated by line colour. Black points and error bars represent (respectively) mean +/− standard deviation of experimental measurements.

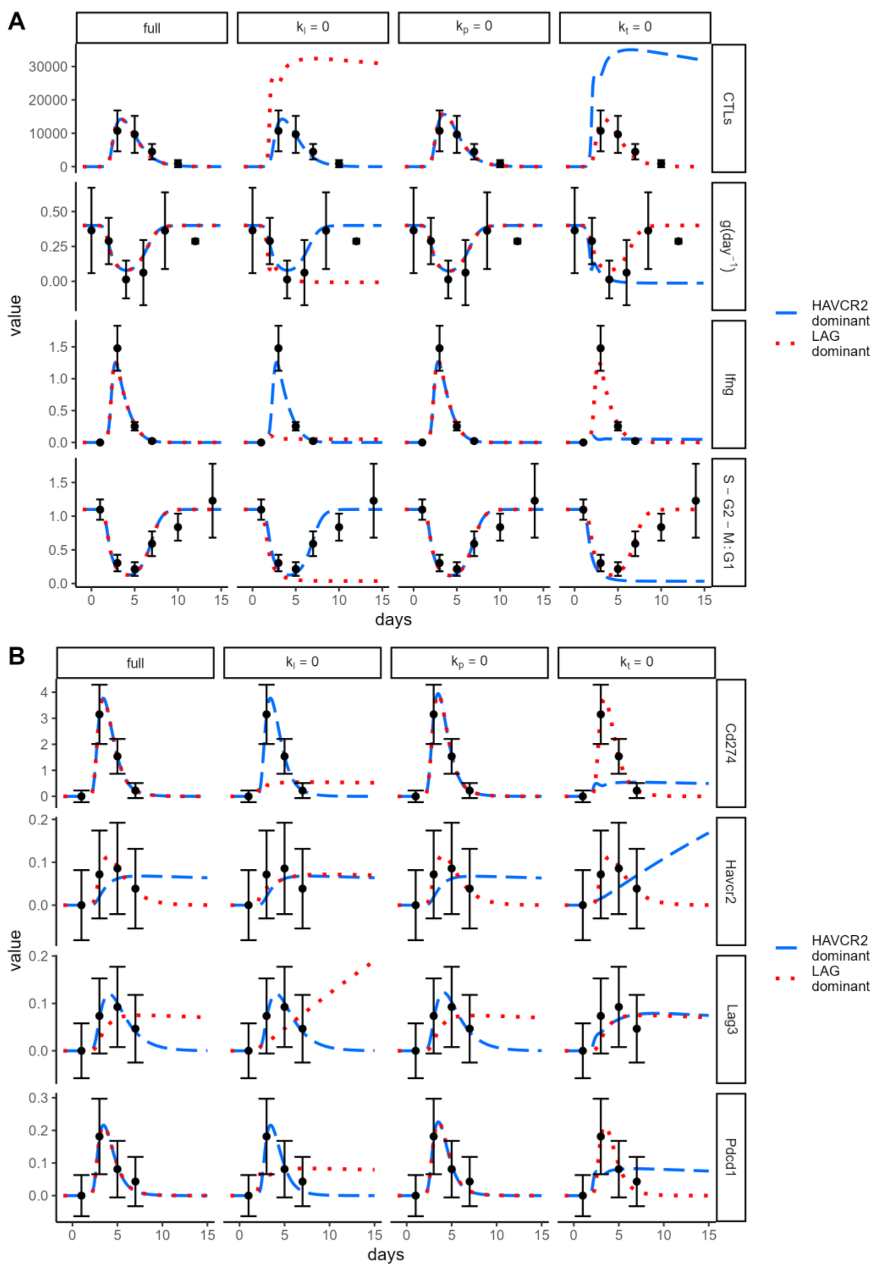

Figure S4. Model comparison with experimental data after disabling subsets of inhibitors, related to Figure 5. A) Model predictions and experimental data for CTL density (top row), volumetric tumour growth (2nd row), *Ifng* mRNA expression (3rd row), or the ratio of S-G_2-M:G_1 nuclei (bottom row). B) Model predictions and experimental data for ICs, with different ICs shown in each row as indicated by facet label. The first column (A-B) shows model predictions for the best-fitting Lag dominant or HAVCR2 dominant parameter sets derived from our optimisation procedure. Subsequent columns show the model output after disabling each of the inhibitors (as indicated by column headers). Disabling LAG3 inhibition significantly disrupts model dynamics for the LAG3 dominant parameter set, whilst disabling HAVCR2 significantly disrupts model dynamics for the HAVCR2 dominant parameter set. In all other cases disabling the immune checkpoints had no noticeable effect on the model outputs. Black points and error bars represent (respectively) mean +/− standard deviation of experimental measurements.

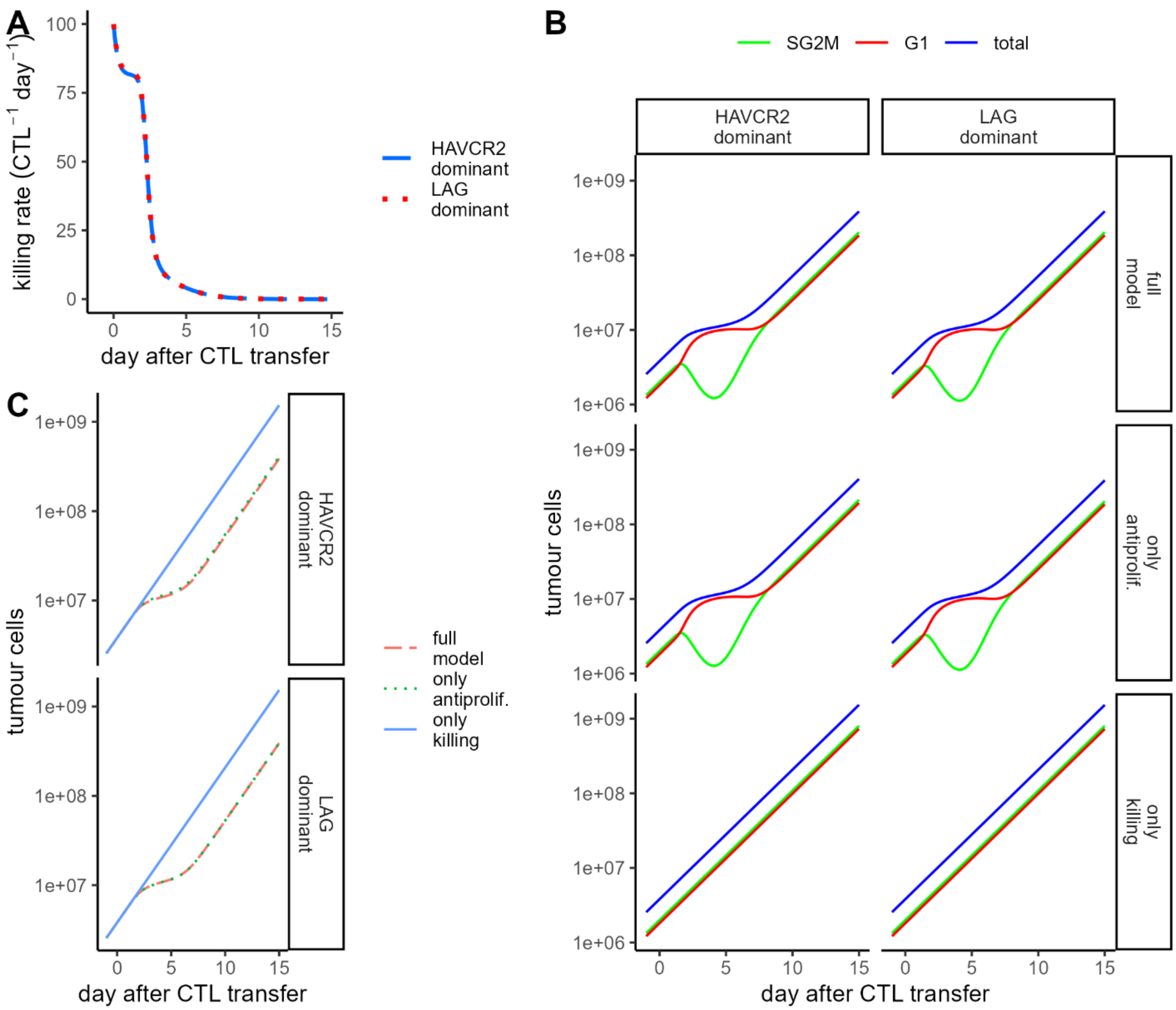

Figure S5. Relative effects of killing and IFNG in the fitted models incorporating immune checkpoints, related to Figure 5. A) Total CTL killing rates predicted using the best fits for LAG-dominant and HAVCR2-dominant parameter sets (represented by indicated colours). B) Comparison of the number of tumour cells, predicted over time for the LAG-dominant and HAVCR2-dominant parameter sets (across columns). Total cells (blue), G_1 phase cells (red), or S-G_2-M phase cells (green) are shown separately for each condition. Across rows are simulations with either all parameters as fitted (top), with killing disabled (middle), or with the antiproliferative effect disabled (bottom). C) Comparison of total number of tumour cells in the model for the 6 conditions simulated in B.

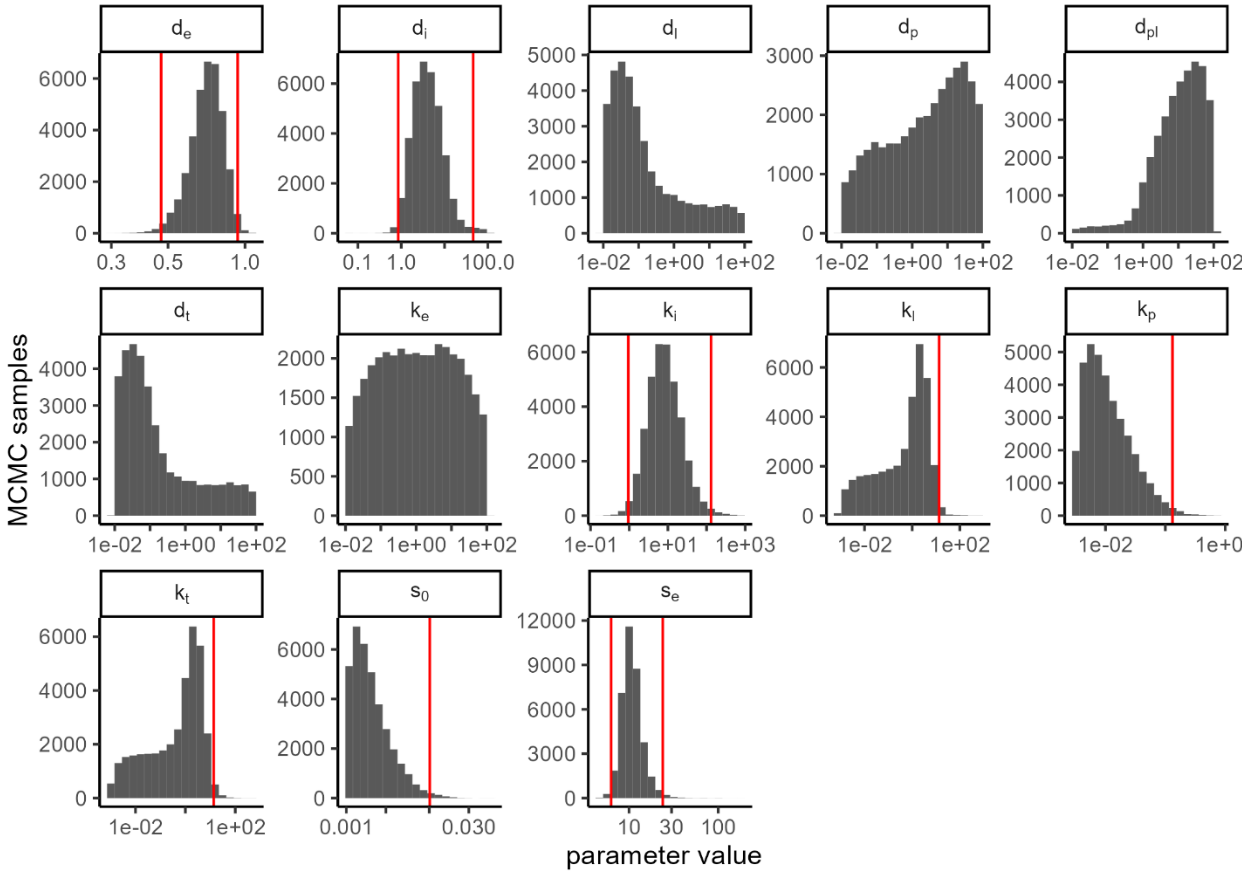

Figure S6. Estimated posterior distribution for each individual parameter, related to Figure 5. Red lines indicate estimated credible intervals determined by the 1-99% quantiles of the posterior distribution. Interval bounds not shown lay outside the range of the prior distribution thus could not be determined. Credible intervals where obtainable are provided in Table 1.

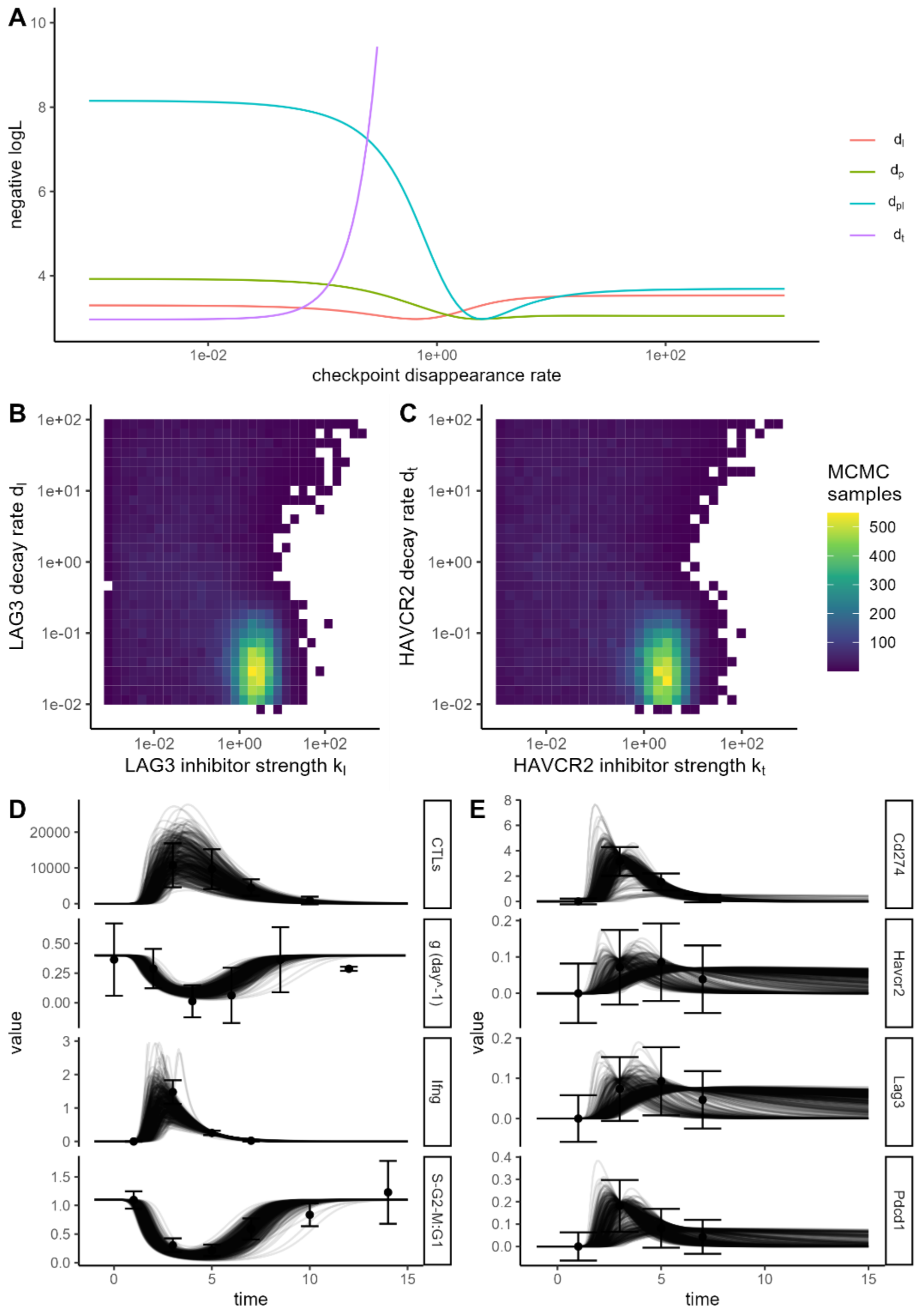

Figure S7. Assessment of parameter identifiability, related to Figure 5. A) Negative log likelihood obtained using the best-fitting (HAVCR2 dominant) parameter set, holding all parameters fixed and varying each indicated parameter. B-C) Estimated joint posterior distributions of parameters d_l and k_l (B) and of d_t and k_t (C). Distributions originate from the values taken by each parameter in the final 50,000 steps of the main Markov chain. D) Estimated posterior prediction densities for CTL density, volumetric tumour growth, *Ifng* mRNA expression, or the ratio of S-G_2-M:G_1 nuclei. E) Estimated posterior prediction densities for ICs, with different ICs shown in each row as indicated by facet label. Posterior prediction density estimates in D-E were obtained by running the model with 500 parameter sets randomly sampled from the final 50,000 steps of the main Markov chain. Black points and error bars in D-E represent (respectively) mean +/− standard deviation of experimental measurements.

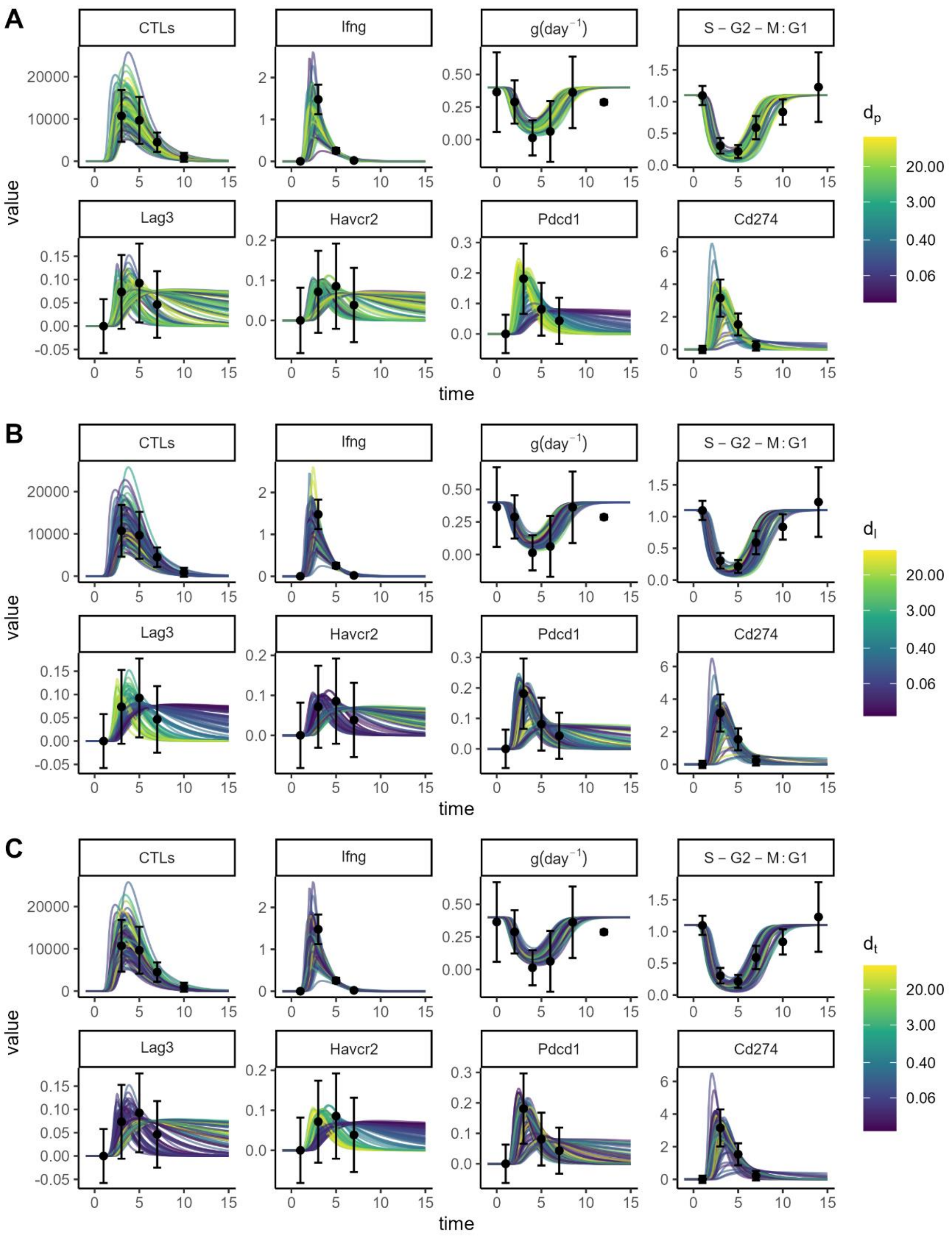

Figure S8. Assessment of trajectories from posterior predictions for CTL density, volumetric tumour growth, *Ifng* mRNA expression, the ratio of S-G_2-M:G_1 nuclei, and different ICs, related to Figure 5. A-C) Estimated posterior prediction densities with trajectories coloured on the basis of parameter values of d_p (A), d_l (B), or d_t (C). Posterior prediction density estimates were obtained by running the model with 100 parameter sets obtained by randomly sampling from the final 50,000 steps of the main Markov chain. Black points and error bars represent (respectively) mean +/− standard deviation of experimental measurements.

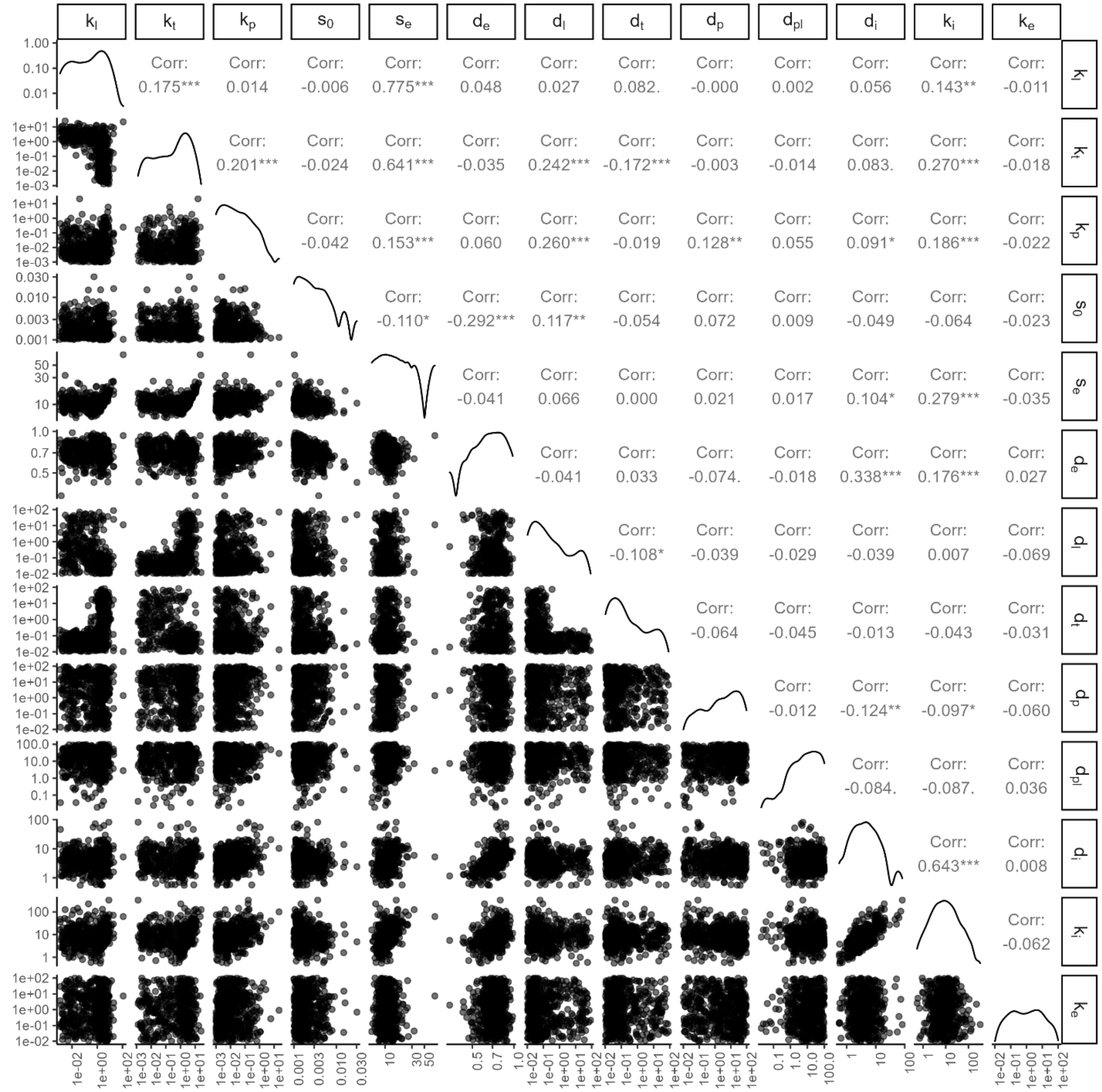

Figure S9. Parameter correlations within posterior distribution, related to Figure 5. Lower-right triangle shows pairwise combinations of fitted parameters. Diagonal panels show the density of each parameter. Upper-right triangle shows correlations between pairwise combinations of fitted parameters. Parameter values are sampled from the values taken by each parameter in the final 50,000 steps of the main Markov chain.
